# TriNet: A tri-fusion neural network for the prediction of anticancer and antimicrobial peptides

**DOI:** 10.1016/j.patter.2023.100702

**Published:** 2023-02-28

**Authors:** Wanyun Zhou, Yufei Liu, Yingxin Li, Siqi Kong, Weilin Wang, Boyun Ding, Jiyun Han, Chaozhou Mou, Xin Gao, Juntao Liu

**Affiliations:** 1 SDU-ANU Joint Science College, Shandong University (Weihai), Weihai 264209, China; 2 School of Mechanical, Electrical & Information Engineering, Shandong University (Weihai), Weihai 264209, China; 3 School of Mathematics and Statistics, Shandong University (Weihai), Weihai 264209, China; 4 Computational Bioscience Research Center (CBRC), Computer, Electrical and Mathematical Sciences and Engineering Division, King Abdullah University of Science and Technology (KAUST), Thuwal 23955, Saudi Arabia

**Keywords:** anticancer peptides, antimicrobial peptides, tri-fusion neural network, training approach

## Abstract

The accurate identification of anticancer peptides (ACPs) and antimicrobial peptides (AMPs) remains a computational challenge. We propose a tri-fusion neural network termed TriNet for the accurate prediction of both ACPs and AMPs. The framework first defines three kinds of features to capture the peptide information contained in serial fingerprints, sequence evolutions, and physicochemical properties, which are then fed into three parallel modules: a convolutional neural network module enhanced by channel attention, a bidirectional long short-term memory module, and an encoder module for training and final classification. To achieve a better training effect, TriNet is trained via a training approach using iterative interactions between the samples in the training and validation datasets. TriNet is tested on multiple challenging ACP and AMP datasets and exhibits significant improvements over various state-of-the-art methods. The web server and source code of TriNet are respectively available at http://liulab.top/TriNet/server and https://github.com/wanyunzh/TriNet.

## Highlights

• Development of TriNet: a deep-learning framework to identify ACPs and AMPs
• TriNet captures serial fingerprint and physicochemical distribution features
• The framework defines three neural networks to process three peptide features
• Development of TVI: an iterative method for training TriNet

## The bigger picture

In drug discovery, the importance of antimicrobial peptides is increasing as multidrug-resistant microbes continue to emerge. In addition, there is a growing clinical interest in anticancer peptides for the treatment of drug-resistant cancer cells. The cost of traditional wet lab experiments to identify such peptides can be significantly reduced by using computational methods that utilize artificial intelligence. In this study, we developed a deep-learning framework called TriNet for the accurate and rapid identification of anticancer and antimicrobial peptides. Benchmarking studies demonstrate that TriNet performs with extensive adaptability and effectiveness in identifying anticancer and antimicrobial peptides. In this work, TriNet is improved through the appropriate constructions of peptide features, the tri-fusion neural network, and the TVI training method. Further refinement may lead to an effective tool for guiding cancer treatment and antibiotic drug design.

## Graphical abstract

**Figure:**
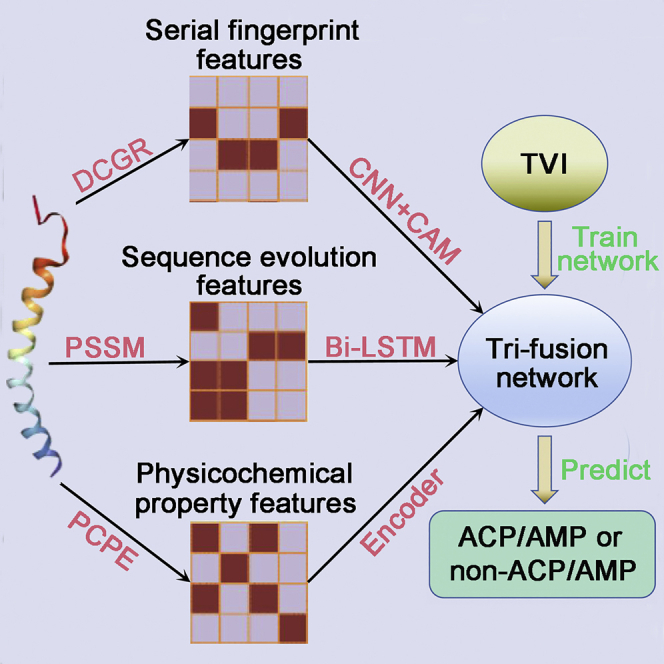

## Teaser

We developed TriNet, a framework for the prediction of anticancer and antimicrobial peptides. The framework captures global sequence and physicochemical distribution information by utilizing three networks, CNN + CAM, Transformer encoder, and Bi-LSTM, which are applied to process these two features and another evolutionary feature. TriNet was trained using TVI, a method developed in this study, and significant improvements are shown in benchmarking studies.

## Introduction

The dramatic increase in antimicrobial resistance poses a severe threat to public health globally.^1^ Due to the misuse or overuse of antibiotic drugs, some bacterial pathogens generate resistance to antimicrobials, which has adverse effects on disease treatments.^2^^,^^3^ Consequently, the discovery of alternative therapies for combating infections caused by multidrug-resistant bacteria is urgently needed.^4^ One promising strategy is to perform therapy based on antimicrobial peptides (AMPs), which can help reduce the likelihood of resistance emergence.^5^

A great number of known AMPs are small molecules with negligible toxicity and broad spectra of activity against bacteria, fungi, viruses, and even cancer cells.^6^^,^^7^ Anticancer peptides (ACPs) are a specific class of AMPs that can control cancer cell resistance to anticancer drugs.^8^ Similar to most AMPs, ACPs with cations can engage electrostatically with the anionic membranes of cancer cells and kill them without destroying normal cells.^9^ In recent years, AMPs, including ACPs, have been widely used for clinical applications in a variety of disease therapies.^10^^,^^11^^,^^12^^,^^13^ Accordingly, the effective identification of peptides with biological activity is crucial for developing candidate drugs. Various experimental and computational methods have been developed. Traditional wet experiments are often expensive and time consuming; hence, the development of reliable computational methods is urgently needed. With the development of artificial intelligence, an increasing number of computational methods based on machine learning have been proposed. For those methods, the extraction of effective peptide sequence features is the critical first step. In recent decades, researchers have explored various algorithms for extracting features from the compositional and distribution information of amino acid sites, and other approaches take advantage of the physicochemical properties that set AMPs or ACPs apart from other peptide sequences. In addition, binary profile features (BPFs),^14^ amino acid composition (AAC), and dipeptide composition (DPC)^15^ are also widely employed. Based on AAC, Chou^16^ proposed the PseAAC model to preserve sequence order information. Wei et al.^17^ proposed an adaptive skip DPC (ASDC) method for enriching DPC features. The compositional-transition-distribution (CTD) algorithm proposed by Dubchak et al.^18^ clusters 20 amino acids into three groups based on specific physicochemical properties and summarizes 21 descriptors containing composition, transition, and distribution information, which can better describe the global compositions of the physicochemical properties of amino acids in peptide sequences.

With the tremendous development of deep learning, in addition to the use of traditional machine learning algorithms, such as support vector machines (SVMs), random forests (RFs), and extreme gradient boosting (XGBoost),^19^ deep-learning techniques, including convolutional neural networks (CNNs) and recurrent neural networks (RNNs), are increasingly being employed by researchers to identify functional peptides. For the prediction of AMPs, Veltri et al.^20^ transformed amino acid residues into 128-dimensional vectors via an embedding layer and combined a convolution layer and a recurrent layer to capture potential sequence information. Su et al.^21^ used multiscale convolutional layers with different filter lengths to capture multiscale motifs in peptide sequences. Fu et al.^22^ proposed a deep neural network (DNN) model called ACEP using convolutional layers and an attention mechanism to fuse the feature tensors generated by a learnable sequence encoding model. For ACP detection, the classical model is the long short-term memory (LSTM) neural network-based deep-learning framework developed by Yi et al. called ACP-DL.^23^ Ahmed et al.^24^ proposed a multiheaded deep CNN model, ACP-MHCNN, for extracting features from different sources of information, such as physicochemical properties and evolutionary information, using parallel CNNs for ACP prediction. Wang et al.^25^ proposed a hybrid CNN-LSTM model termed CL-ACP that applies a CNN to focus on local information and an LSTM to extract the dependencies of residues. Lv et al.^26^ used two kinds of sequence-embedding technologies, SSA and UniRep, related to DNNs based on LSTM to complete classification tasks.

The existing methods for predicting ACPs and AMPs mainly have the following shortcomings. In terms of feature extraction, the features of peptide sequence residues are usually extracted in a one-by-one manner in most existing predictors. Thus, the global information on peptide sequences cannot be captured. Methods such as ACEP, which uses attention scores to capture relationships across peptides, should be adopted. In addition, in existing methods, several physicochemical properties are usually selected directly from hundreds of properties,^14^ which may result in serious redundancy or low quality of the chosen properties. In terms of the design of neural networks for processing extracted features, many models fail to design specific neural networks based on the properties of different features and even apply the same or similar neural network architectures to process different kinds of features. Without effective peptide feature processing, the performance of existing methods still has plenty of room for improvement. In terms of neural network training, the training and validation sets are randomly separated in traditional training approaches. Thus, there is no guarantee that hard samples (samples that are very likely to be wrongly predicted) are well trained, since they may be totally split into the validation set with no or only a few samples in the training set. In recent years, several partition approaches, including SPXY,^27^ Rank-KS,^28^ and SPXYE,^29^ have been proposed to split training and validation sets. The core idea of these methods is to repeatedly select samples with the maximal distance until a predefined number of samples is obtained. Then, the selected and remaining samples are regarded as training and validation sets, respectively. However, in all of these methods, the separations are performed prior to training, ignoring the possibility that different neural networks have different hard samples. Therefore, more appropriate feature extraction methods, neural networks, and separations of training and validation sets are urgently needed to improve the identification of ACPs and AMPs.

**Figure 1:**
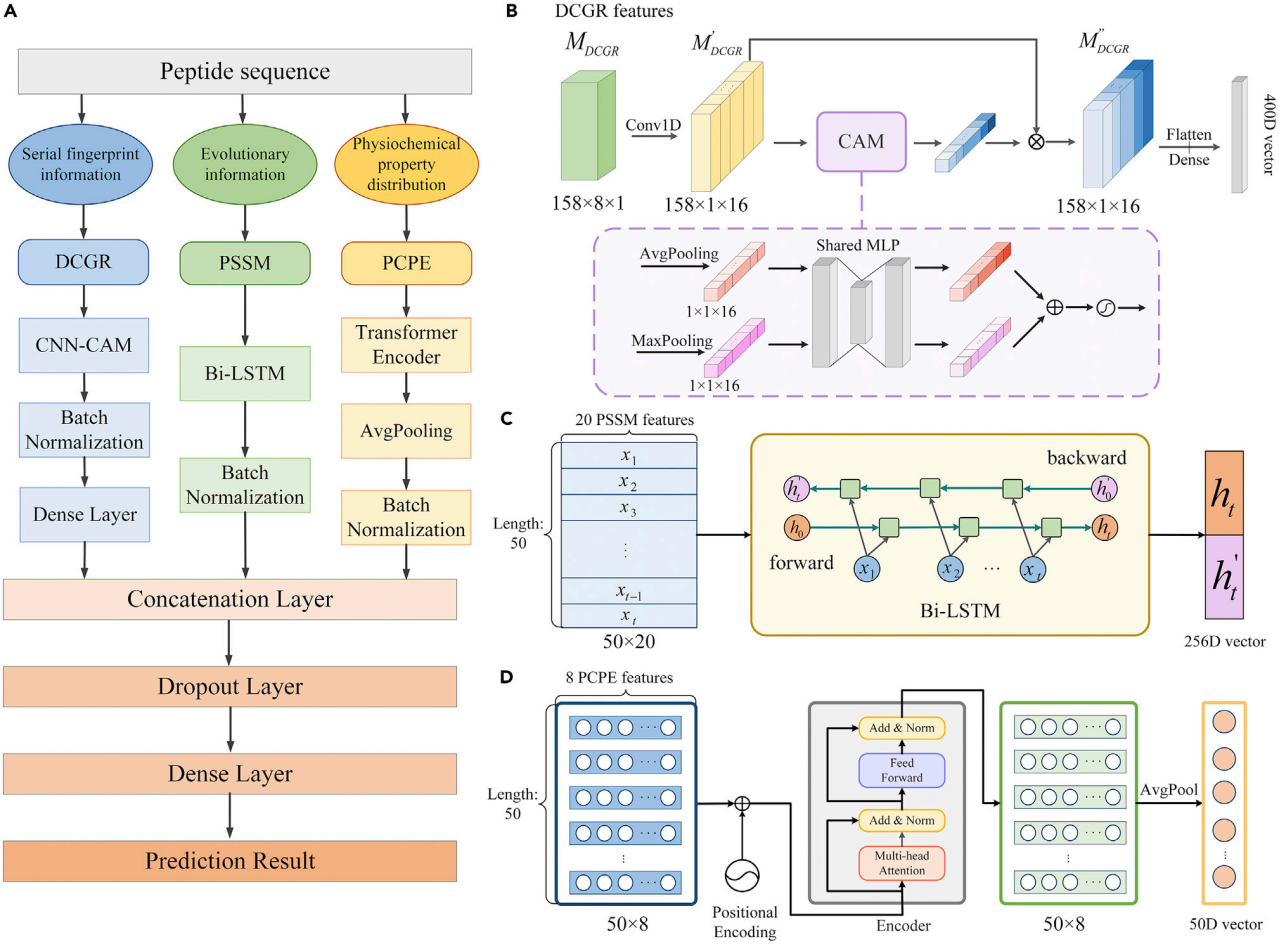
Overall structure of TriNet (A) Flowchart of the proposed TriNet model. (B) Architecture of the DCGR-CNN-CAM mechanism. First, a matrix *M*_*DCGR*_ containing serial fingerprint information is fed into a convolutional layer, and a feature map *M′*_*DCGR*_ is generated. Then, a CAM layer is conducted on *M′*_*DCGR*_ to obtain the channel weights, and the weight-assigned feature map *M″*_*DCGR*_ is flattened and passed through a dense layer. (C) Architecture of the PSSM-Bi-LSTM module. Given a feature matrix *M*_*PSSM*_, a Bi-LSTM network is applied to process the sequence evolution features. (D) Architecture of the PCPE-encoder module. The feature matrix *M*_*PCPE*_ is first fed into the encoder block of a transformer by using positional encoding, and then average pooling is applied after the encoder module.

In this study, we introduce TriNet, a tri-fusion neural network for ACP and AMP prediction (see Figure 1 for the workflow of TriNet). (1) TriNet is designed based on the assumption that whether a peptide is an ACP or AMP should be determined by multiple pieces of information and their effective fusion. (2) In addition to the frequently used position-specific scoring matrix (PSSM) feature, TriNet introduces another two features for representing the information contained in the serial fingerprint and physicochemical properties of a peptide sequence. (3) TriNet employs three parallel networks, a channel attention module (CAM) based on convolutional layers (for processing serial fingerprint features), a bidirectional LSTM network (Bi-LSTM; for processing the sequence evolution features), and an encoder module (for processing physicochemical property features), attempting to effectively fuse the above three kinds of features. (4) Different from traditional neural network training methods, TriNet is trained by a training approach termed TVI to achieve a better training effect, which is achieved by iterative interactions between the samples in the training and validation datasets to generate more appropriate training and validation sets based on the biases of neural networks.

We benchmarked TriNet on multiple challenging ACP and AMP datasets by using both cross-validation and independent testing, and the results showed that the proposed framework achieved substantially improved performance over that of other ACP/AMP prediction tools. In addition, we fully evaluated the effectiveness of the TVI training method for the prediction of both ACPs and AMPs, and in multiple other network models, and found that TVI effectively reconstructed the most appropriate training and validation sets based on the biases of a given neural network. Finally, we tested the effectiveness of the three proposed features and network structures on all six datasets, and the results clearly demonstrated the extensive adaptability and effectiveness of the extracted features and the network structures. TriNet has been proven to be very sensitive in detecting ACPs and AMPs, demonstrating its great potential for guiding the development of small-peptide drugs targeting cancer cells or other pathogens, such as bacteria, fungi, and viruses.

## Results

TriNet is a framework for predicting ACPs/AMPs based on peptide sequences by effectively fusing the information contained in the serial fingerprints, sequence evolutions, and physicochemical properties of peptide sequences and then training the network with a training method called TVI. We first tested the effectiveness of the TVI training method by comparing it with the traditional training method (random sampling). Then, we evaluated the performance of TriNet on a diverse set of challenging datasets and compared it with six other ACP prediction algorithms, ACP-DL,^23^ MHCNN,^24^ iACP-DRLF,^26^ CL-ACP,^25^ DeepACPpred,^30^ and AntiCP 2.0,^31^ as well as six AMP prediction algorithms, DNN,^20^ APIN,^21^ ACEP,^22^ CAMP-RF, CAMP-SVM, and CAMP-ANN.^32^ Finally, we analyzed the effectiveness of the extracted features as well as the structures of TriNet. In this study, the accuracy, sensitivity, specificity, precision, F1 score, and Matthews correlation coefficient (MCC) metrics were employed as evaluation criteria (see the experimental procedures).

### Performance evaluation of the TVI training method

**Figure 2:**
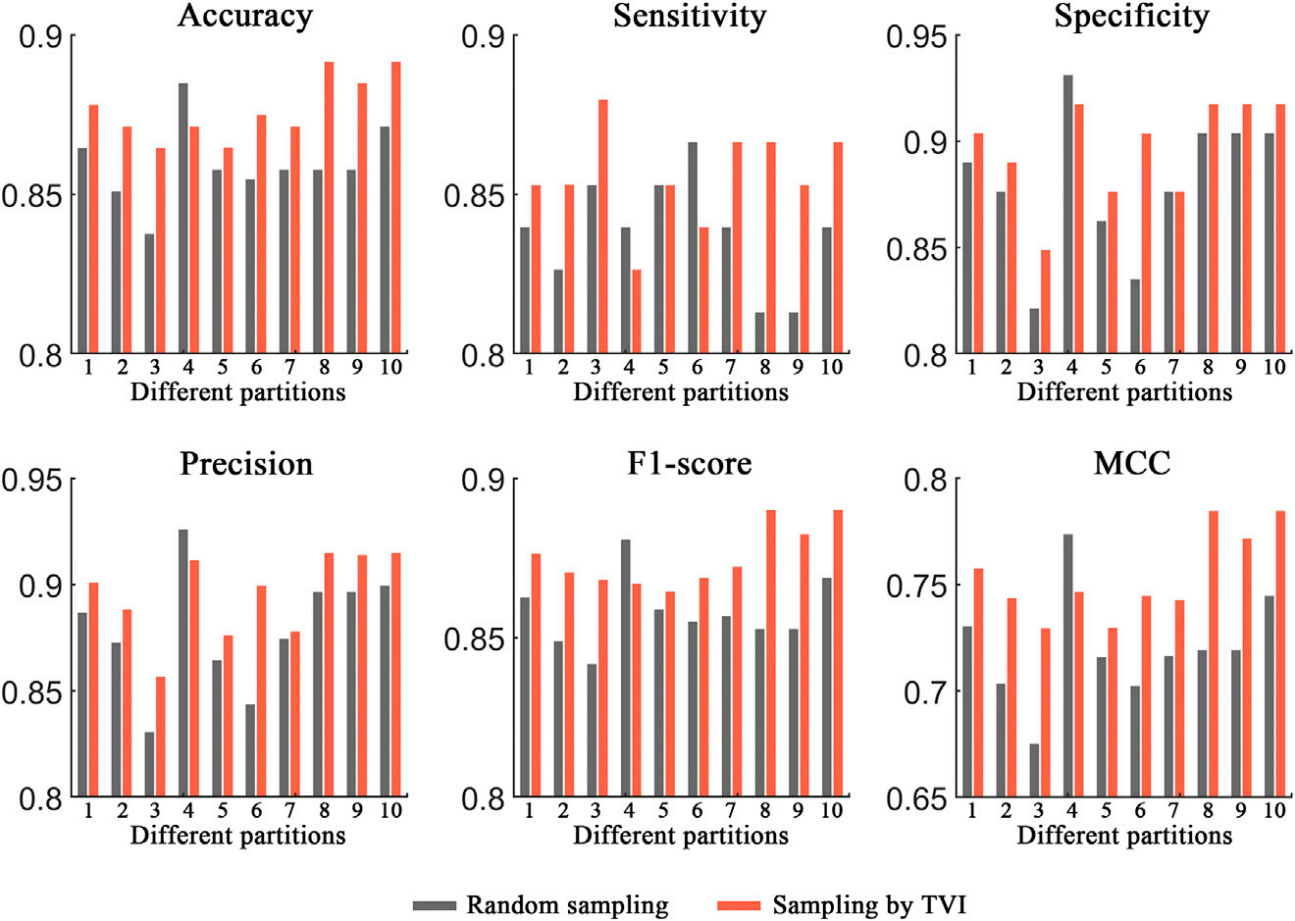
Performance comparison between the traditional training approach and the TVI method on the ACP740 dataset Six different evaluation metrics are shown: accuracy, sensitivity, specificity, precision, F1 score, and MCC.

**Figure 3:**
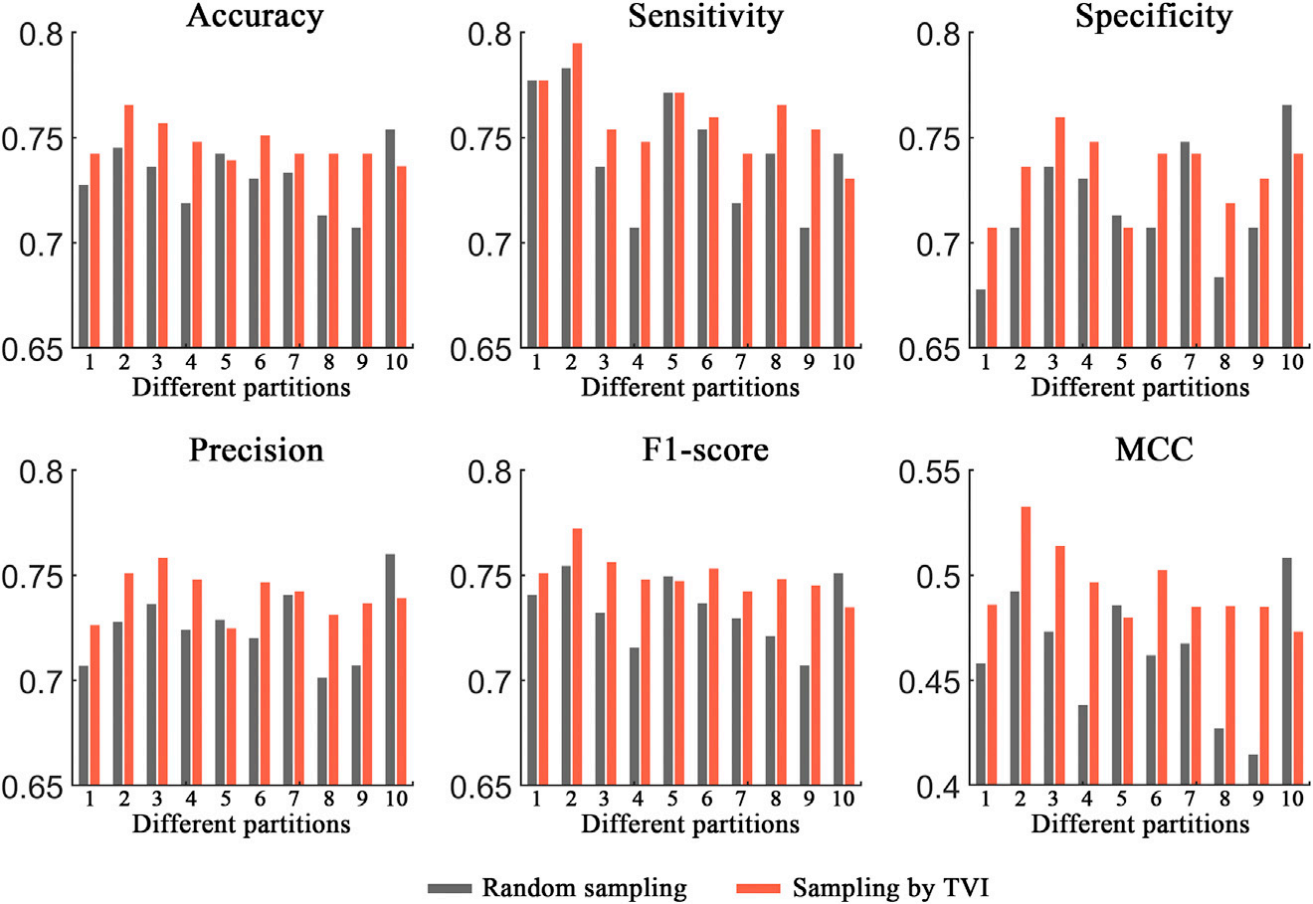
Performance comparison between the traditional training approach and the TVI method on the ACPmain dataset Six different evaluation metrics are shown: accuracy, sensitivity, specificity, precision, F1 score, and MCC.

**Figure 4:**
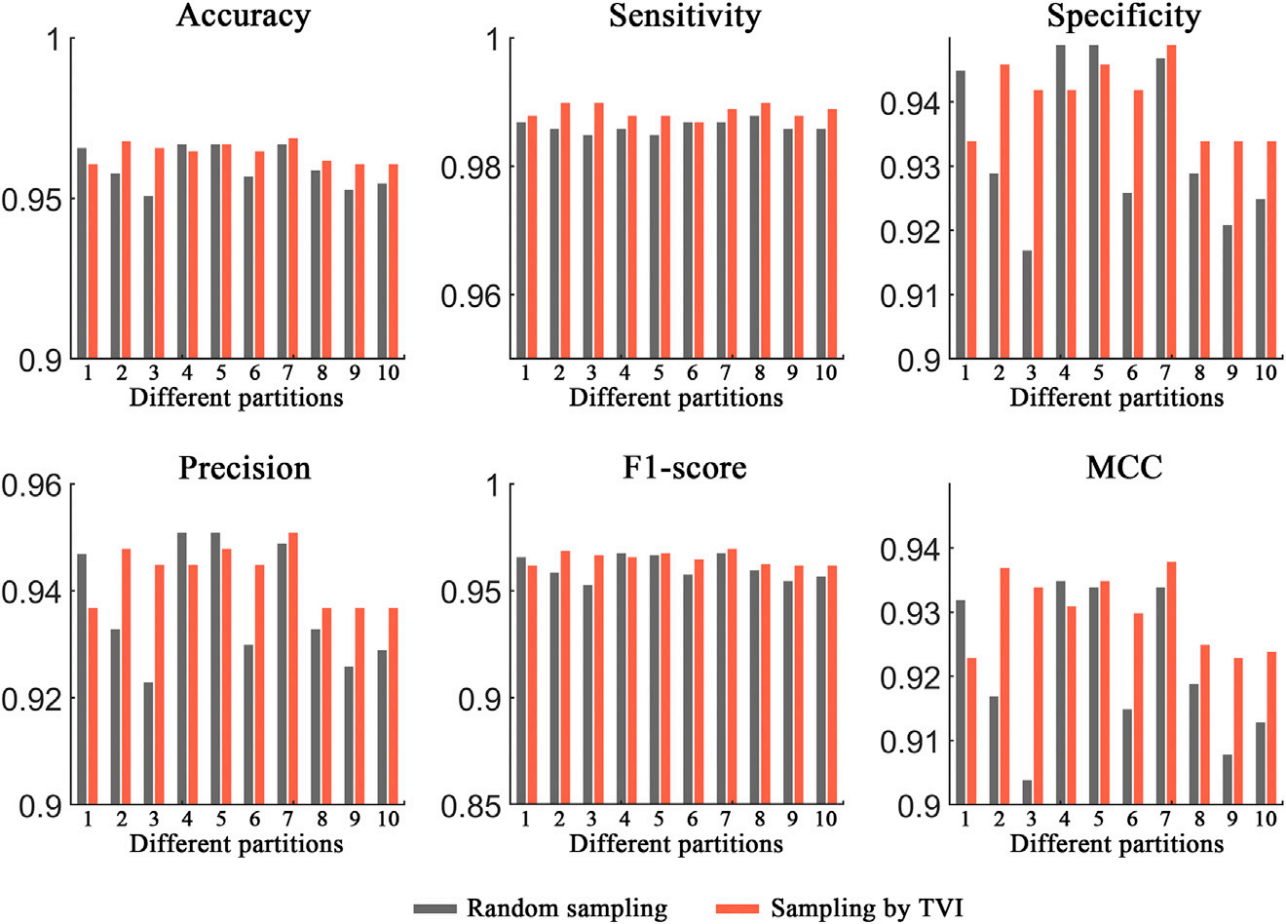
Performance comparison between the traditional training approach and the TVI method on the Xiao dataset Six different evaluation metrics are shown: accuracy, sensitivity, specificity, precision, F1 score, and MCC.

In this section, two ACP datasets (ACP740 and ACPmain) and an AMP dataset (Xiao dataset) were used to evaluate the effectiveness of the TVI training method, and the process was as follows. For ACP740, 20% of the ACPs and non-ACPs were randomly selected as the fixed test set, and the remaining 80% of the samples were then randomly separated into a training set (containing 473 samples) and a validation set (containing 119 samples). The random separation of the training and validation sets was performed 10 times, and the 10 different pairs of training and validation sets produced were used for network training and validation, respectively. Then, different trained models were evaluated on the test set, and the results showed that the network models demonstrated obvious biases on different separations of the training and validation sets (see Figures 2, 3, and 4; random sampling). For example, the performance differences between the two separations were 4.7%, 5.3%, 11.0%, 9.5%, 3.9%, and 0.098 in terms of the accuracy, sensitivity, specificity, precision, F1 score, and MCC metrics, respectively, on the ACP740 dataset. On the ACPmain dataset, the differences reached 4.7%, 7.6%, 8.8%, 5.9%, 4.7%, and 0.094, respectively. The differences were 1.6%, 0.3%, 3.2%, 2.8%, 1.5%, and 0.031, respectively, on the Xiao dataset.

For comparison purposes, the TVI method was also tested on the 10 training and validation set separations generated by random sampling, and it performed better than the traditional training approach, with average improvement rates of 2.0%, 2.1%, 1.9%, 1.9%, 2.0%, and 4.6% in terms of the accuracy, sensitivity, specificity, precision, F1 score, and MCC metrics, respectively, on the ACP740 dataset (see Figure 2). On the ACPmain dataset, the average improvement rates were 2.0%, 2.1%, 1.9%, 1.9%, 2.0%, and 4.7%, respectively (see Figure 3). The average improvement rates reached 0.47%, 0.24%, 0.72%, 0.63%, 0.45%, and 0.98%, respectively, on the Xiao dataset (see Figure 4). Moreover, the largest improvement rates in terms of the accuracy, sensitivity, specificity, precision, F1 score, and MCC metrics were 3.9%, 6.6%, 8.2%, 6.6%, 4.4%, and 9.1% on the ACP740 dataset; 5.0%, 6.6%, 5.0%, 4.3%, 5.4%, and 16.9% on the ACPmain dataset; and 1.6%, 0.3%, 3.2%, 2.8%, 1.5%, and 3.3% on the Xiao dataset, respectively.

Moreover, for the TVI training method, we calculated the performance differences between each of the two separations of the training and validation sets and found that the differences obviously decreased (see Table S1). The results demonstrated that the TVI method effectively reduced the biases of the tested network models on different separations of the training and validation sets.

In addition, we evaluated the effectiveness of TVI on other ACP network models. Two models, ACP-DL and MHCNN, were employed to perform the test because they provided full codes that could be utilized to implement our TVI training method. After the evaluation was completed, the results demonstrated that the two network models still exhibited obvious biases on different separations of the training and validation sets, and the TVI training method still performed better than the traditional training method, suggesting its strong generalization ability (see Notes S1 and S2 and Figures S1–S4). The detailed test information of each model on different datasets can be seen at https://github.com/wanyunzh/TriNet.

**Figure 5:**
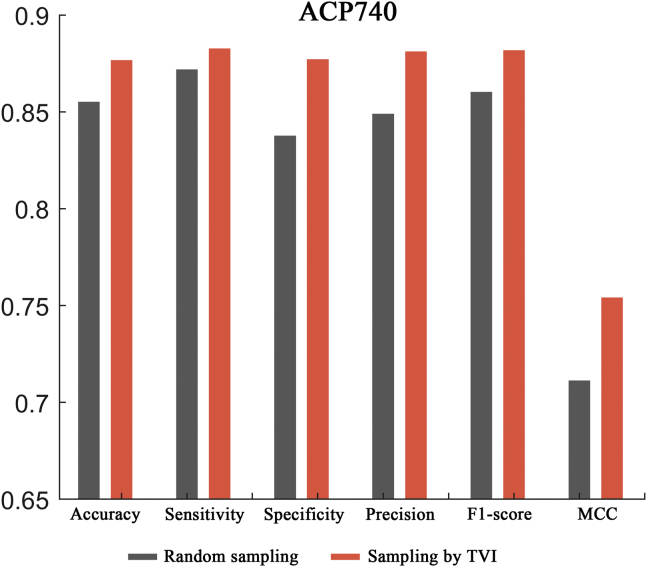
Comparison between the traditional training approach and the TVI method conducted via 5-fold cross-validation on the ACP740 dataset The comparison was performed under six different evaluation metrics: accuracy, sensitivity, specificity, precision, F1 score, and MCC.

Furthermore, to prevent the influence of the fixed test sets, a 5-fold cross-validation was also performed on the ACP740 dataset by using the TVI method. As shown in Figure 5, the TVI method consistently performed better than the traditional training approach, with improvement rates of 2.5%, 1.2%, 4.7%, 3.8%, 2.5%, and 6.0% in terms of the accuracy, sensitivity, specificity, precision, F1 score, and MCC metrics, respectively, on the ACP740 dataset, indicating that the TVI training method was not restricted to the test sets. Based on the above facts, we believe that the TVI method exhibits great potential for improving the performance of peptide predictions.

### Performance comparison with other existing models

#### Comparison with ACP predictors

In this section, we compared the performance of TriNet with that of several other state-of-the-art ACP predictors by conducting 5-fold cross-validations on the ACP740 dataset and independent tests on the ACPmain and ACPalternate datasets. On the ACP740 dataset, we compared TriNet with ACP-DL, MHCNN, iACP-DRLF, CL-ACP, and DeepACPpred.^23^^,^^24^^,^^25^^,^^26^^,^^30^ On the ACPmain and ACPalternate datasets, we compared it with ACP-DL, MHCNN, iACP-DRLF, and AntiCP 2.0.^23^^,^^24^^,^^26^^,^^31^ In these ACP prediction approaches, to our knowledge, a validation set is not established during model training when an independent test is performed. Harrington^33^ indicated that a single split of the training and test sets can result in an inaccurate evaluation of the tested model’s performance. Therefore, we randomly selected 20% of the peptides from the ACPmain and ACPalternate training datasets to form the validation sets. For a fair comparison, all the compared methods were retrained by using the same training and validation sets on the two datasets and then tested on the independent test sets.

**Figure 6:**
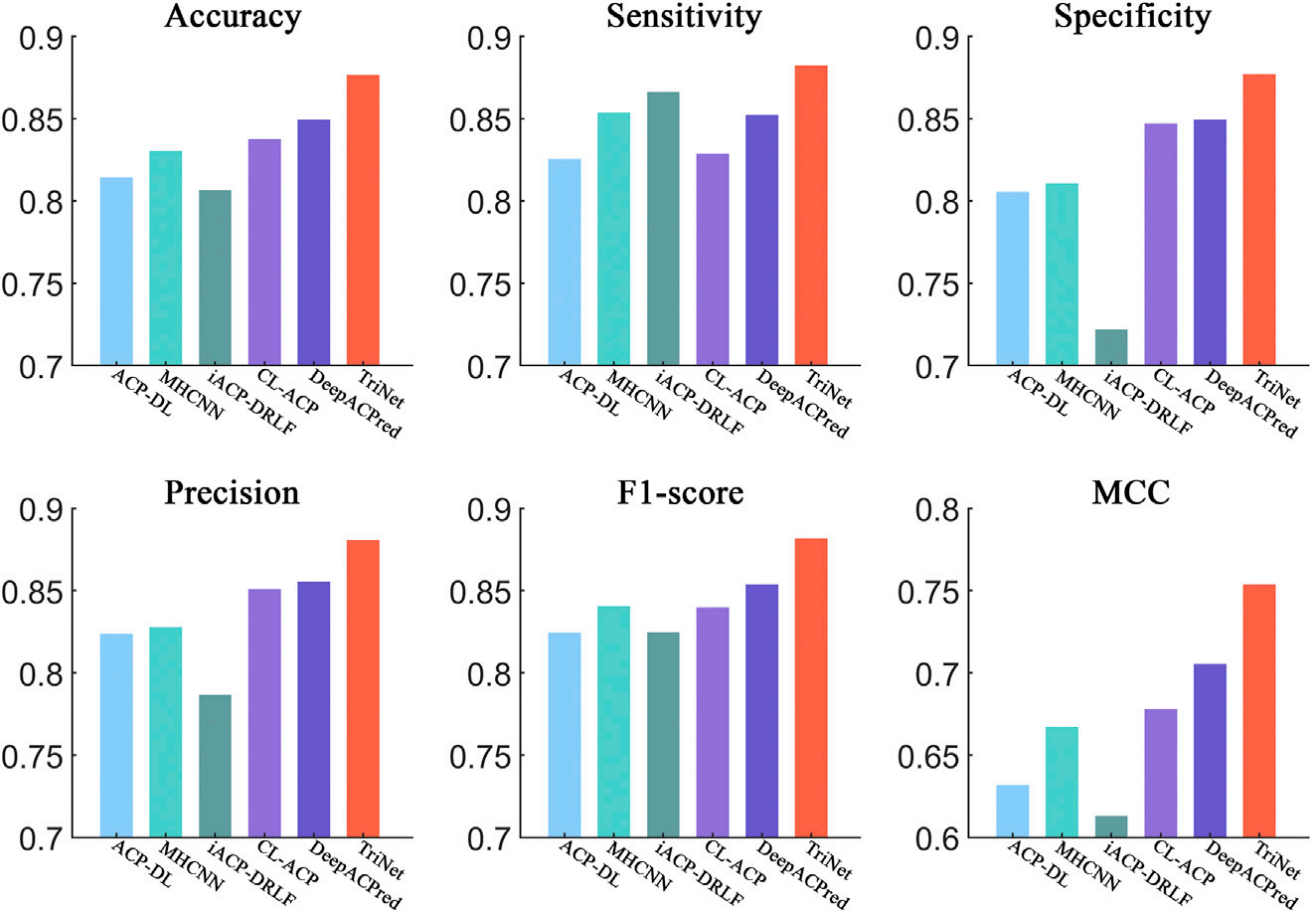
Comparison of TriNet with existing models on the ACP740 dataset using 5-fold cross-validation Six different evaluation metrics are shown: accuracy, sensitivity, specificity, precision, F1 score, and MCC.

**Figure 7:**
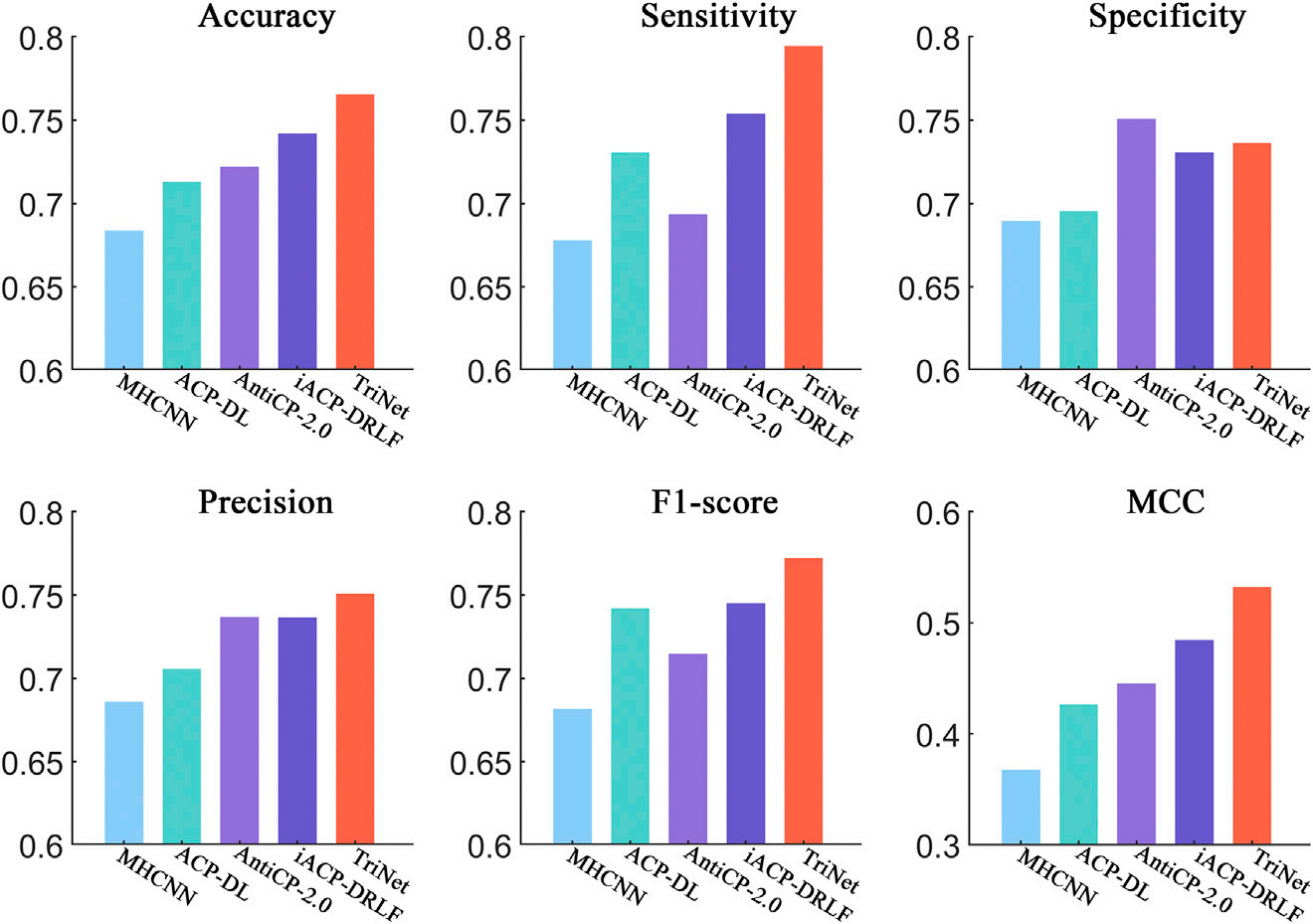
Comparison of TriNet with existing models on the ACPmain independent dataset Six different evaluation metrics are shown: accuracy, sensitivity, specificity, precision, F1 score, and MCC.

**Figure 8:**
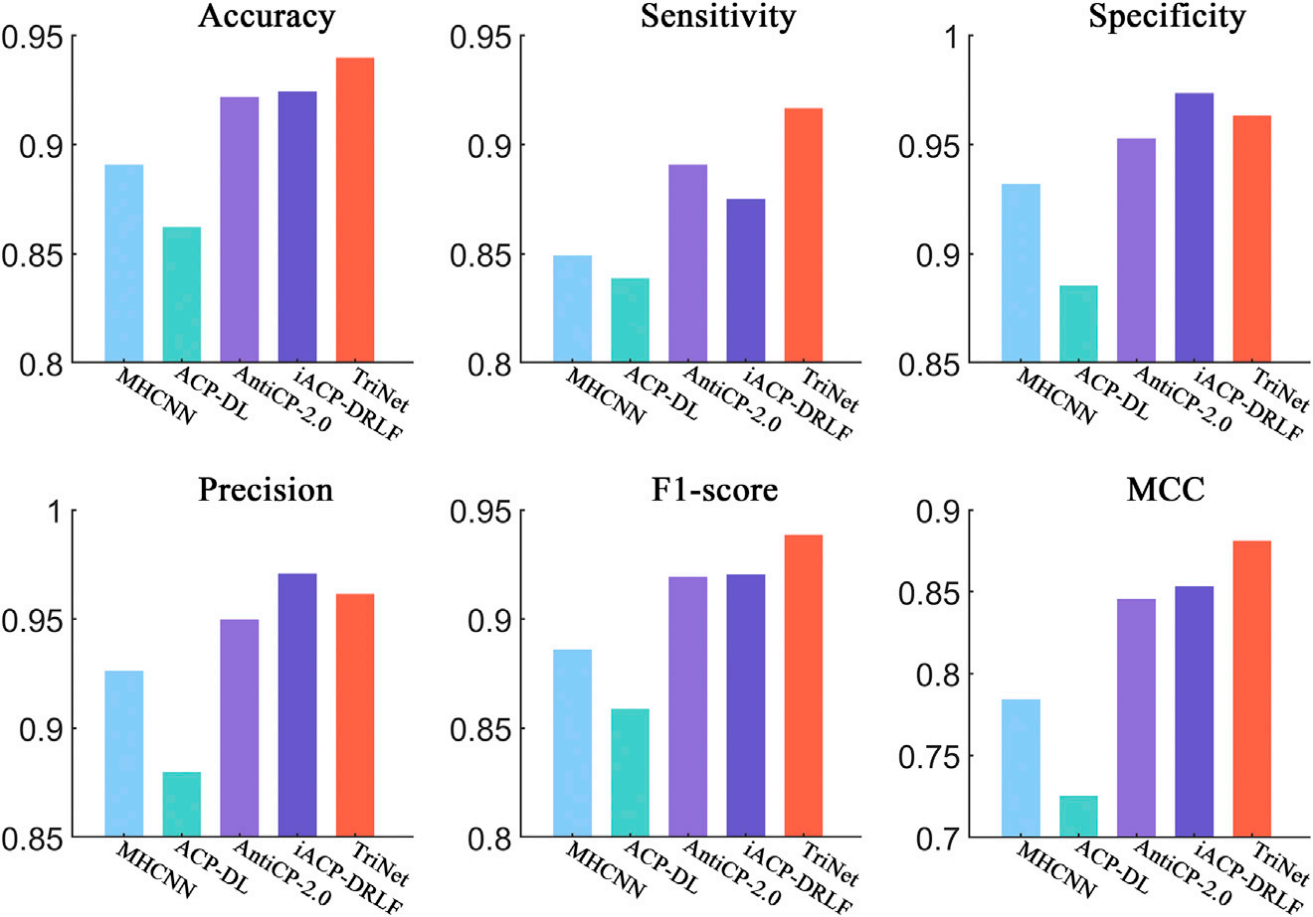
Comparison of TriNet with existing models on the ACPalternate independent dataset Six different evaluation metrics are shown: accuracy, sensitivity, specificity, precision, F1 score, and MCC.

After comparison, the results showed that TriNet performed the best among all the compared methods on all three datasets. In detail, the improvement rates achieved by TriNet over the other compared methods were 3.2%–8.6%, 1.9%–6.9%, 3.2%–21.5%, 3.0%–12.0%, 3.2%–6.9%, and 6.9%–22.9% on the ACP740 dataset (see Figure 6) in terms of the accuracy, sensitivity, specificity, precision, F1 score, and MCC metrics, respectively). The improvement rates in comparison with other methods were 3.2%–12.0%, 5.4%–17.2%, 1.9%–9.5%, 3.6%–13.2%, and 9.8%–44.7% on the ACPmain independent dataset in terms of accuracy, sensitivity, precision, F1 score, and MCC metrics, respectively (see Figure 7), and 1.7%–9.0%, 2.9%–9.3%, 2.0%–9.3%, and 3.3%–21.4% on the ACPalternate independent dataset in terms of accuracy, sensitivity, F1 score, and MCC metrics, respectively (see Figure 8). It should be noted that, although the specificity of AntiCP-2.0 is slightly higher than that of TriNet on the ACPmain independent dataset, its other indicators are lower than those of TriNet. Similarly, on the ACPalternate independent dataset, the specificity and precision of iACP-DRLF are slightly higher than those of TriNet, while its other indicators are lower than those of TriNet. Therefore, TriNet demonstrated the best overall performance on all three ACP datasets.

#### Comparison with AMP predictors

**Figure 9:**
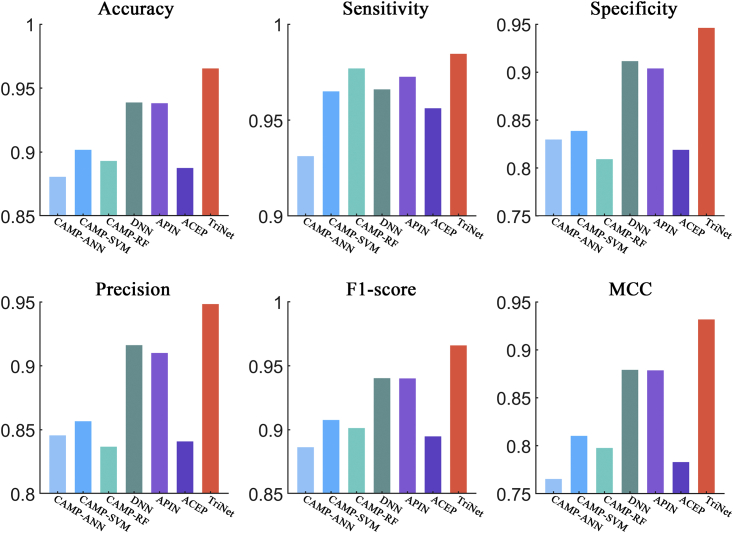
Comparison of TriNet with existing models on Xiao’s independent dataset Six different evaluation metrics are shown: accuracy, sensitivity, specificity, precision, F1 score, and MCC.

**Figure 10:**
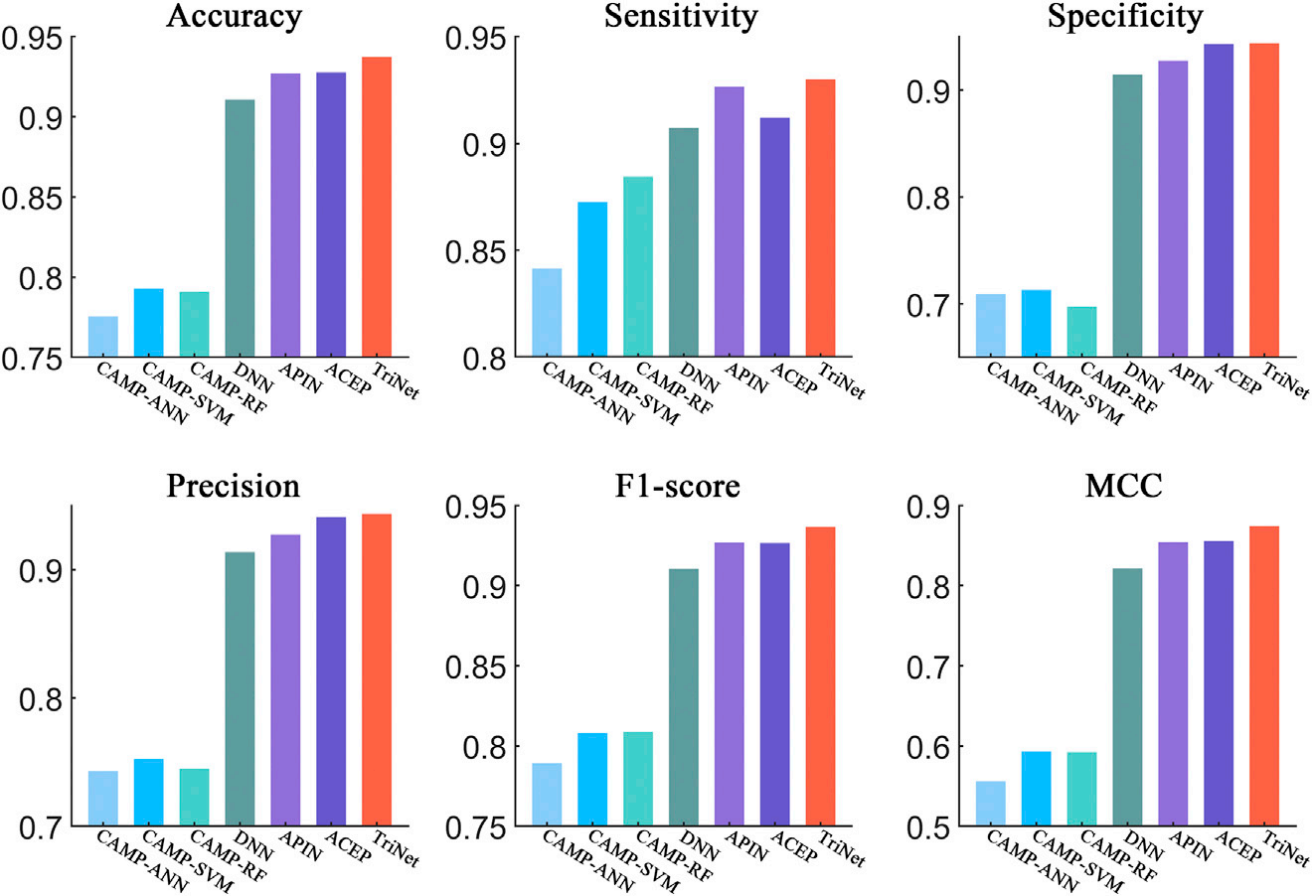
Comparison of TriNet with existing models on the AMPlify dataset Six different evaluation metrics are shown: accuracy, sensitivity, specificity, precision, F1 score, and MCC.

**Figure 11:**
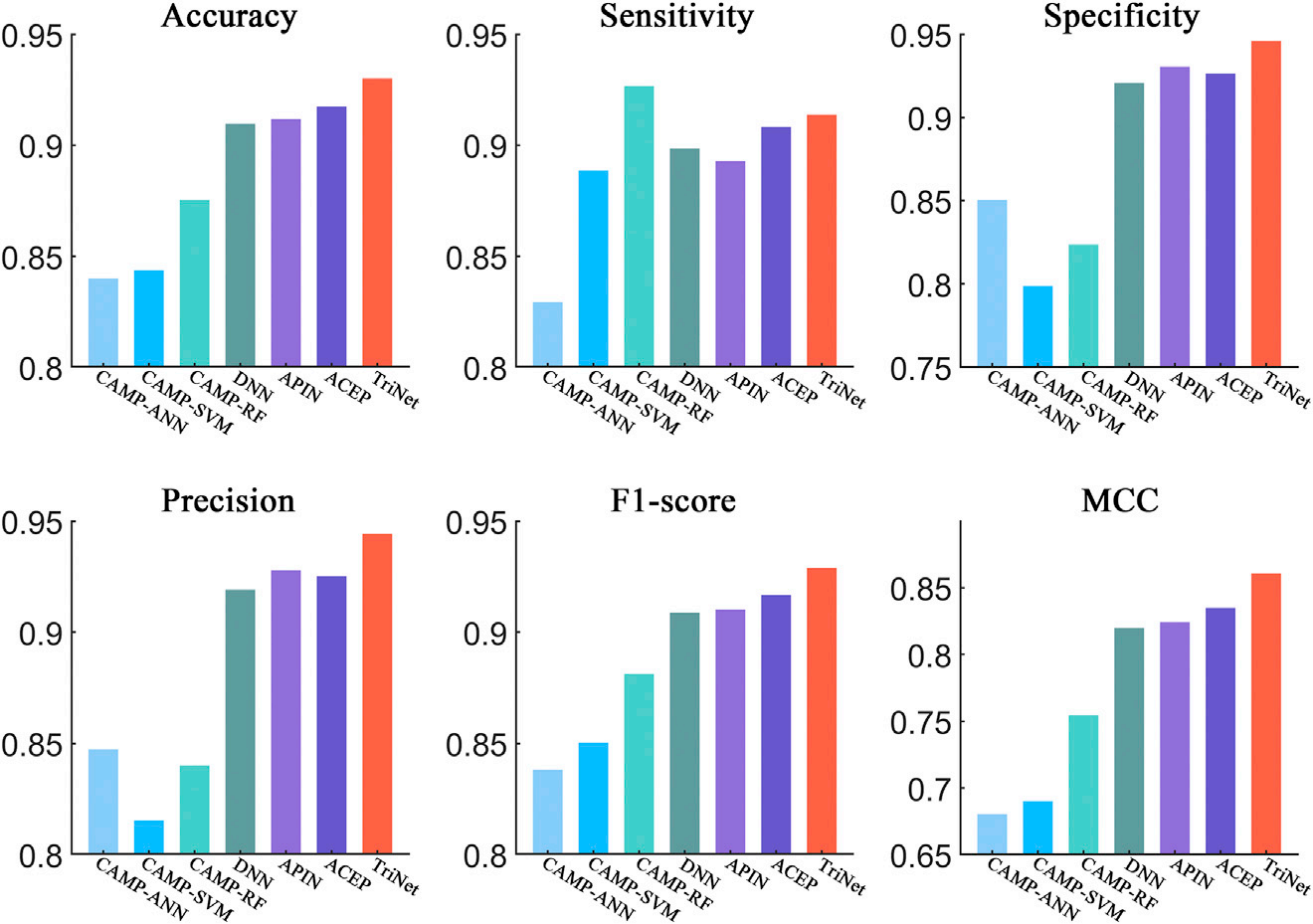
Comparison of TriNet with existing models on the DAMP dataset Six different evaluation metrics are shown: accuracy, sensitivity, specificity, precision, F1 score, and MCC.

In addition to ACP predictors, we compared TriNet with AMP prediction tools, including DNN, APIN, ACEP, CAMP-RF, CAMP-SVM, and CAMP-ANN,^20^^,^^21^^,^^22^^,^^32^ by testing them on three AMP datasets. After comparison, the results showed that TriNet performed better than all the compared methods on the three datasets. In detail, the improvement rates achieved by TriNet over the other compared methods were 2.8%–9.6%, 0.78%–5.7%, 3.8%–16.9%, 3.5%–13.3%, 2.7%–9.0%, and 6.0%–21.7% on the Xiao independent dataset (see Figure 9) in terms of the accuracy, sensitivity, specificity, precision, F1 score, and MCC metrics, respectively, and 1.0%–20.8%, 3.9%–10.5%, 0.13%–26.9%, 0.23%–26.9%, 1.1%–18.7%, and 2.2%–57.2% on the AMPlify dataset (see Figure 10), respectively. On the DAMP dataset (see Figure 11), the improvement rates in terms of accuracy, specificity, precision, F1 score, and MCC were 1.4%–10.7%, 1.7%–18.4%, 1.8%–15.8%, 1.3%–10.8%, and 3.1%–26.5%. Although the sensitivity of TriNet is lower than that of CAMP-RF, its other indicators are much higher, demonstrating that the best overall performance is achieved using TriNet (see Figure 11).

### Evaluation of the effectiveness of the extracted features and the network structures

In this paper, we carried out multiple tests on the three datasets, ACP740, ACPmain, and Xiao to verify the effectiveness of our feature extraction methods as well as the superiority of the network structures.

#### Effectiveness of the extracted features

In this section, we first demonstrated the advantages of the improved DCGR method over the original DCGR approach in terms of extracting the sequence serial fingerprint features. Then, we verified the importance of combining all three features. Finally, we demonstrated the extensive adaptability and effectiveness of the three features.

To demonstrate the advantages of the improved DCGR method, we compared its performance with that of the original DCGR approach on all three datasets, and the results showed that the improved method performed better than the original techniques in terms of all the metrics on the three datasets (see Table S2). Then, we attempted to verify the importance of combining all three features by removing each feature individually, and the results showed that the loss of any of the three features resulted in performance degradation on all three datasets (see Table S3). In addition, in comparison with the physicochemical property feature, the removal of the serial fingerprint or sequence evolution feature caused a more serious performance decline. Finally, to demonstrate the extensive adaptability and effectiveness of the three features, we replaced the neural network with the XGBoost^34^ algorithm, which is a popular traditional machine learning technique, for retraining the samples on all three datasets. In detail, the three feature matrices obtained from DCGR, PSSM, and physicochemical property embedding (PCPE) were first flattened and then concatenated to generate a feature vector for XGBoost. The testing results (see Tables S4–S6) showed that XGBoost achieved higher performance than many of the compared models on all three datasets, demonstrating the extensive adaptability and effectiveness of the features extracted in this study.

#### Effectiveness of the network structures

Regarding the CNN-CAM mechanism for processing the serial fingerprint feature, we replaced the 1 × 8 kernel with three frequently used square kernels with sizes of 2, 3, and 5, and the results showed that our property-based 1 × 8 kernel performed the best (see Table S7), mainly because such a kernel size made the network learn the shared weights based on each physicochemical property. The CAM contains two pooling strategies: average pooling and maximum pooling. We first replaced this module with the squeeze-and-excitation network (SENet),^35^ which uses only global average pooling, and the prediction performance obviously decreased on all three datasets (see Table S8). As shown in previous studies,^36^ maximum pooling compensates for the global information gained from average pooling by reflecting the salient part of each channel. Then, we replaced the CAM with another popular convolutional block attention module (CBAM)^36^ that has a spatial attention module (SAM) after its CAM, and we found that the prediction performance still declined on most datasets (see Table S8). Since the SAM mainly focuses on the importance of the spatial features, the serial fingerprint feature extracted by DCGR had no spatial or positional properties.

For the encoder module, we tested different numbers of heads in the self-attention module. In detail, we set 1, 2, and 4 heads and compared the resulting performances. The results showed that the single-head self-attention mechanism performed better than the multihead self-attention mechanism (see Table S9). The reason for this may be that a single head is able to make the network cover the most effective information concerning the distribution of the physicochemical properties. As a consequence, the use of multiple heads makes the model fail to capture the differences among the heads, and finally, the multihead models become more complex and ineffective.

## Discussion

In this study, we introduced TriNet, a tri-fusion neural network for ACP or AMP prediction. After evaluating the performance of TriNet and comparing it with other leading prediction methods on multiple challenging datasets, we found that TriNet demonstrated much higher accuracy in terms of predicting both ACPs and AMPs than all the compared methods under commonly used criteria. The superiority of TriNet may be attributed to the following method innovations.

First, we proposed that a prediction method for ACPs and AMPs should effectively fuse multiple pieces of information, based on which the TriNet framework was designed. Second, in addition to the frequently used sequence evolution feature, we introduced another two features, the serial fingerprint and physicochemical property features, which appropriately characterize the global sequence information and the distributions of the physicochemical properties of peptides. The test results demonstrated the extensive adaptability and effectiveness of the proposed features. Third, based on the properties of the three features, we specifically designed three network structures, which appropriately processed each of the features and then effectively fused them for the final predictions. Fourth, we developed a neural network training approach called TVI, which was able to generate more appropriately separated training and validation sets based on the biases of a network model. In addition, we provided the learning curves of all six datasets (see Figures S6–S11) to demonstrate the degree of overfitting, and it was shown that there is no obvious overfitting phenomenon on any of these datasets.

In supervised deep-learning fields, setting both the validation and the test sets is of great importance for evaluating the generalization ability of a network model according to the predictive power of blind test sets. However, as we know, in the field of ACP prediction, many models have only training and test sets, which clearly leads to information leakage from the test set and an inaccurate evaluation of the model’s performance. In contrast, we set the validation sets for both 5-fold cross-validation and independent testing.

Despite the obvious advantages of TriNet, we still have a long way to go to completely solve the ACP/AMP prediction problem, and further improvements can still be made on TriNet in the future. For example, the current model is not an end-to-end model. Thus, it still takes some time to calculate the corresponding features. Therefore, the inference time of TriNet may be longer than that of end-to-end frameworks. In addition, we note that the current version of TVI may perform slightly worse than traditional training methods in some cases, and more attention should be given to the following issues. (1) How can the starting epoch for interaction among the samples in the training and validation sets be determined? (2) How can the number of interacting samples between the two sets be determined? (3) How can the interaction termination epoch be determined? Moreover, the issue of determining whether interactions are required for the given separation of the training and validation sets still needs to be further investigated. The future version of TriNet will attempt to solve these problems and make further improvements.

The results of the evaluations showed that our method could clearly distinguish between ACPs/AMPs and non-ACPs/AMPs, and the potential of TriNet for identifying ACPs/AMPs will help researchers develop small-peptide drugs targeting cancer cells or other pathogens, such as bacteria, fungi, and viruses. In addition, the TVI training method may become the next trend for training different neural networks in other areas.

## Experimental procedures

### Resource availability

#### Lead contact

The lead contact for questions about this paper is Juntao Liu, who can be reached at juntaosdu@126.com.

#### Materials availability

No unique materials were generated from this study.

### Methodology

#### Dataset preparation

In this study, six datasets were collected to test the prediction performance of TriNet, including three ACP datasets (ACP740 dataset, ACPmain dataset, and ACPalternate dataset) for ACP prediction and three AMP datasets (Xiao dataset, DAMP dataset, and AMPlify dataset) for AMP prediction. ACP740 was introduced by Yi et al.^23^; it contains 376 experimentally validated ACPs and 364 AMPs without anticancer activity, and the sequence similarity between each pair of peptides is no greater than 90%. ACPmain and ACPalternate were introduced from Agrawal et al.,^31^ and each dataset contains two subsets. The first subset of ACPmain, which includes 689 experimentally validated ACPs and 689 non-ACPs, was separated into two parts for training and validation with a 4:1 ratio. The second subset of ACPmain, which includes 172 experimentally validated ACPs and 172 non-ACPs, was used as the independent test set. The first subset of ACPalternate, which includes 776 experimentally validated ACPs and 776 non-ACPs, was also separated into two parts for training and validation with a 4:1 ratio. The second subset of ACPmain, which includes 194 experimentally validated ACPs and 194 non-ACPs, was used as the independent test set.

For the AMP datasets, Xiao’s benchmark training dataset^37^ comprises 1,388 AMPs and 1,440 non-AMPs, and the corresponding independent test set comprises 920 AMPs and 920 non-AMPs. However, the dataset from Xiao^37^ has a major difference in length distribution between AMP and non-AMP sequences. We followed the same method as Veltri et al.^20^ and randomly adjusted the lengths of non-AMP sequences to more closely resemble AMP sequences to avoid learning the length differences. The AMP and non-AMP sequence length distributions of the original dataset and our readjusted dataset are provided in Figures S12 and S13. Then, Xiao’s training dataset was divided into two parts for training and validation with a 4:1 ratio. DAMP was introduced by Veltri et al.,^20^ and the 3,556 peptide sequences (1,778 AMPs and 1778 non-AMPs) with a similarity of no more than 40% were divided into three parts: 1,424 for training, 708 for validation, and 1,424 for testing. AMPlify was introduced by Li et al.,^38^ and the non-AMP sequences in the dataset were also adjusted to match the length distributions of the AMP sequences. The training dataset comprising 3,338 AMPs and 3,338 non-AMPs was also divided into two parts for training and validation with a 4:1 ratio, and the independent test set comprises 835 AMPs and 835 non-AMPs.

#### Overview of the TriNet framework

The TriNet pipeline was designed to predict ACPs and AMPs based solely on the given peptide sequences. In this study, we assumed that whether a peptide was an ACP or AMP could be predicted by effectively combining three kinds of features representing the serial fingerprints, sequence evolutions, and physicochemical properties of peptide sequences. Therefore, the main architecture of the TriNet pipeline comprises three parallel components, a CNN-CAM, a Bi-LSTM network, and an encoder module, for processing and fusing the above three features (Figure 1). By using batch normalization, 400-, 256-, and 50-dimensional feature vectors were obtained as the outputs of each branch. These feature vectors were then concatenated and passed through dropout and dense layers to generate the final prediction results. The final dense layer employs a sigmoid function generating a score in [0,1] to determine that the peptide is an ACP/AMP if the score is no smaller than 0.5 and a non-ACP/AMP otherwise.

Moreover, to obtain better training results than those of the traditional training method, which randomly separated the training and validation datasets, a training approach termed TVI was designed to reseparate the training and validation sets based on the structure of the neural network, which was achieved through iterative interaction of the samples in the training and validation datasets. In the following sections, we introduce each part of the TriNet method in detail.

#### Extraction of peptide sequence features

Given a peptide sequence, three kinds of features reflecting the information of the serial fingerprints, sequence evolutions, and physicochemical properties of peptide sequences were extracted as follows.

*Extracting the serial fingerprint features of sequences*. DCGR^39^ is a protein sequence feature extraction method based on chaotic game representation (CGR)^39^^,^^40^ that attempts to capture the global characteristics of a protein sequence; therefore, the extracted features can effectively reflect the serial fingerprint information of the given peptide sequences (see Note S3 for the method details). The original DCGR method obtained distance matrices only in four quadrants, and the information between the points that crossed quadrants was therefore lost. To recover this lost information, we improved the DCGR method by rotating the coordinate axis by 45° to obtain another four distance matrices (see Figure S5). Then, for each CGR curve, eight distance matrices, *A*_*i*1_, *A*_*i*2_, …, *A*_*i*8_, could be calculated, and the final 158 × 8 feature matrix *M*_*DCGR*_ for each peptide could be expressed as:

(Equation 1) $$d_{i}={\left[\rho \left(A_{i1}\right),\rho \left(A_{i2}\right),\ldots ,\rho \left(A_{i8}\right)\right]}^{T}\text{,}$$

(Equation 2) $$M_{DCGR}={\left[d_{1},d_{2},\ldots ,d_{i},\ldots ,d_{158}\right]}^{T}\text{,}$$

where *ρ*(*A*_*ij*_) denotes the leading eigenvalues of the distance matrix *A*_*ij*_ and 158 represents the 158 physicochemical properties selected from the AAindex.

*Extracting sequence evolution features*. The PSSM is frequently applied to detect distant homologs using iterations.^41^^,^^42^ An element (*i*, *j*) in the PSSM is proportional to the probability of the residue at position *i* being replaced by amino acid *j*, reflecting the evolutionary information of peptide sequences. The PSI-BLAST^43^ tool was employed to obtain an *L* × 20 feature matrix *M*_*PSSM*_ for each peptide.

*Extracting sequence physicochemical property features*. PCPE is capable of reflecting the distributions of physicochemical properties in peptide sequences. Regarding the choice of physicochemical properties, traditional methods usually select specific physicochemical properties directly and therefore may result in the chosen physicochemical properties exhibiting redundancy or low quality. In contrast, we first employed the method proposed by Saha et al.^44^ to group the 556 physicochemical properties into eight clusters and extracted the most representative property in each cluster to obtain more comprehensive physicochemical properties while avoiding redundancies. Then, by using PCPE, each amino acid was encoded into an eight-dimensional vector, and an *L* × 8 feature matrix *M*_*PCPE*_ was finally constructed for each peptide.

To make the feature matrices *M*_*PSSM*_ and *M*_*PCPE*_ of all the peptides with different lengths have the same dimensions, we set the sequence length *L* to 50 and used zero-padding for peptides whose lengths were less than 50.

#### Processing of the serial fingerprint features via the CNN-CAM module

The feature matrix *M*_*DCGR*_ obtained from DCGR was reshaped into a three-dimensional tensor and fed into an improved CNN (see Figure 1B), which is capable of capturing important features through local connectivity and weight sharing. Traditional CNNs usually apply square kernels to learn to convolve feature matrices. However, each row of the feature matrix *M*_*DCGR*_ denotes one of the 158 physicochemical properties, and the columns represent the eight features extracted from one CGR curve. Therefore, more appropriate kernels of size 1 × 8 (instead of the frequently used square kernels) were applied by TriNet to learn the shared weights for each of the 158 properties. The number of filters was set to 16 in this study.

The CNN effectively captured the local information from each physicochemical property, based on which the CAM^36^ was then employed to obtain the global information by emphasizing important features from all 158 properties. The CAM model is able to emphasize more valuable features by assigning larger weights. In detail, after calculating the three-dimensional feature map *M′*_*DCGR*_ *= f*_*conv*_ (*M*_*DCGR*_) from the convolutional layer, each channel *C*_*i*_ of *M′*_*DCGR*_ was assigned a channel weight *CAM*_*i*_ according to the classification importance of this channel. First, global average and maximum pooling were performed on the feature map *M′*_*DCGR*_, followed by a shared multilayer perceptron (MLP) comprising two dense layers. The whole process of the CAM can be formulated as follows:

(Equation 3) $$CAM\left({M^{\prime }}_{DCGR}\right)=\sigma \left\{MLP\left[AvgPool\left({M^{\prime }}_{DCGR}\right)\right]+MLP\left[MaxPool\left({M^{\prime }}_{DCGR}\right)\right]\right\}=\sigma \left\{W_{1}\left[W_{0}\left({M^{\prime }}_{avg}\right)\right]+W_{1}\left[W_{0}\left({M^{\prime }}_{\max}\right)\right]\right\}\text{,}$$

where *σ* denotes the sigmoid function, *M′*_*DCGR*_∈*R*^158×1×16^ and *M′*_*avg*_, *M′*_*max*_∈*R*^1×1×16^ are two matrices that calculate the average and maximum pooling, respectively, and *W*_0_∈*R*^2×16^ (with the rectified linear unit [ReLU] activation function) and *W*_1_∈*R*^16×2^ represent the weight matrices of the shared MLP.

The channel weights were then assigned to the corresponding channels of the feature map *M′*_*DCGR*_ for element-wise multiplication, and the weight-assigned feature map *M″*_*DCGR*_∈*R*^158×1×16^ was generated. Then, *M″*_*DCGR*_ was flattened and passed through a dense layer and transformed into the final DCGR feature vector *F*_*DCGR*_ with 400 dimensions.

#### Processing of the sequence evolution features via the Bi-LSTM layer

As shown in Figure 1C, the feature matrix *M*_*PSSM*_ obtained from the PSSM was fed into the Bi-LSTM layer. Different from the traditional RNN, the LSTM network^45^ is able to learn and capture both the long- and the short-term dependencies among the amino acids of a peptide sequence. Moreover, studies have shown that certain types of residues are usually favored at the N terminus and C terminus of ACPs and AMPs, which play crucial roles in identifying ACPs and AMPs.^15^^,^^46^ Therefore, by analyzing the peptide sequences in the forward and backward directions, Bi-LSTM is capable of obtaining information from the C terminus and N terminus for peptides with lengths of no more than 50 amino acids at the same timestep. The calculations of the forward LSTM can be summarized as follows:

(Equation 4) $$f_{t}=\sigma \left(W_{hf}\;h_{t-1}+W_{xf}\;x_{t}+b_{f}\right)\text{,}$$

(Equation 5) $$i_{t}=\sigma \left(W_{hi}\;h_{t-1}+W_{xi}\;x_{t}+b_{i}\right)\text{,}$$

(Equation 6) $${\tilde{c}}_{t}=\tanh\;\left(W_{hc}\;h_{t-1}+W_{xc}\;x_{t}+b_{c}\right)\text{,}$$

(Equation 7) $$O_{t}=\sigma \;\left(W_{ho}\;h_{t-1}+W_{xo}\;x_{t}+b_{o}\right)\text{,}$$

(Equation 8) $$c_{t}=f_{t}⊗c_{t-1}+i_{t}⊗{\tilde{c}}_{t}\text{,}$$

(Equation 9) $$h_{t}=O_{t}⊗\tanh\;\left(c_{t-1}\right)\text{,}$$

where *t* = 1, 2, …, 50 represents the order of 50 amino acids of a peptide sequence; *W*_*hf*_, *W*_*hi*_, *W*_*hc*_, *W*_*ho*_, *W*_*xf*_, *W*_*xi*_, *W*_*xc*_, and *W*_*xo*_ are weight matrices; *b*_*f*_, *b*_*i*_, *b*_*c*_, and *b*_*o*_ are bias vectors; *f*_*t*_ is the forget gate; *i*_*t*_ is the input gate; *o*_*t*_ is the output gate; *x*_*t*_ is the current input; *c*_*t-*1_ is the previous cell state; *c*_*t*_ is the current cell state; ${\tilde{c}}_{t}$ is the value added to the cell state; *h*_*t-*1_ and *h*_*t*_ are the previous and current hidden states, respectively; and ⊗ represents the elementwise multiplication operations.

The backward LSTM works in the same way as the forward LSTM with the calculated current hidden state being *h′*_*t*_. The final PSSM feature vector is then formulated as *F*_*PSSM*_ = [*h*_*t*_, *h′*_*t*_] of 256 dimensions, with *t* being the last time step.

#### Processing the physicochemical property features via the encoder module

The feature matrix *M*_*PCPE*_ obtained from PCPE was fed into the encoder block, with each row representing an eight-dimensional embedding vector (see Figure 1D). The encoder block was designed based on the encoder of a transformer,^47^ which contains multihead self-attention mechanisms, a feedforward network, and skip connections followed by layer normalization. The main part of the transformer is multihead self-attention, which is able to calculate the dependencies between amino acid residues despite the long distances between them, hence efficiently capturing the dependency information of the physicochemical properties of specific peptides. In this paper, single-head self-attention was employed, and its calculation process is summarized as follows:

(Equation 10) $$q_{i}=W_{q}\;p_{i},k_{i}=W_{k}\;p_{i},\nu_{i}=W_{y}\;p_{i},i=1,2,\ldots ,L\text{,}$$

(Equation 11) $$Q={\left[q_{1},q_{2},\ldots ,q_{L}\right]}^{T},K={\left[k_{1},k_{2},\ldots ,k_{L}\right]}^{T},V={\left[\nu_{1},\nu_{2},\ldots ,\nu_{L}\right]}^{T}\text{,}$$

(Equation 12) $$Attention\;\left(Q,K,V\right)=Softmax\;\left(A\right)\;V=Softmax\;\left[Q\;K^{T}/Sqrt\;\left(d_{k}\right)\right]\;V\text{,}$$

where *A* is the attention score matrix; *q*_*i*_, *k*_*i*_, and *v*_*i*_ are query, key, and value vectors, respectively; *d*_*k*_ is their dimensionality; and *W*_*q*_, *W*_*k*_, and *W*_*v*_∈ $R^{d_{k}\times d_{p}}$ are the corresponding weight matrices.

Furthermore, since the order of the residues plays a crucial role in a peptide sequence, positional encoding, using the sine and cosine to reflect the distribution of the physicochemical properties in a peptide sequence, was applied in this study as follows:

(Equation 13) $$PE\;\left(pos,2i\right)=\sin\;\left({pos/10000}^{2i/d_{p}}\right)\text{,}$$

(Equation 14) $$PE\;\left(pos,2i+1\right)=\cos\;\left({pos/10000}^{2i/d_{p}}\right)\text{,}$$

where *pos* represents the positions of the amino acids in the sequence, 2*i* and 2*i* + 1 denote the even and odd element sites in the embedding vectors, respectively, and *d*_*p*_ = 8 is the dimensionality of the embedding vectors.

Then, a feature matrix *M′*_*PCPE*_ = [*p*_1_, *p*_2_ …, *p*_L_]^*T*^ obtained by adding the positional encoding information was constructed, with *p*_*i*_ denoting the feature vector of the *i*-th residue and *L* = 50 representing the sequence length. Passing through the encoder module, average pooling was applied, and the final 50-dimensional PCPE feature vector *F*_*PCPE*_ was calculated for each peptide.

#### Network training by iterative interaction between the training and validation sets

After constructing a neural network, traditional training methods usually randomly separate the training and validation sets and then train the model on the training set and validate it on the validation set. In fact, neural networks may show great biases on different separations, and therefore, different separations of the training and validation sets may largely influence the training of the network model and hence the performance achieved on the testing set. To construct more appropriate training and validation sets by considering the biases of a specific neural network, a method termed TVI was proposed by iteratively interacting the samples in the training and validation sets as follows.

Step 1. Randomly separate a training set *T* = {(*x*_1_, *y*_1_), (*x*_2_, *y*_2_), …, (*x*_*n*_, *y*_*n*_)} and a validation set *V* = {(*x′*_1_, *y′*_1_), (*x′*_2_, *y′*_2_), …, (*x′*_*m*_, *y′*_*m*_)}, where *x*_*i*_ and *x′*_*i*_ are the feature vectors of the samples in the training and validation sets, respectively, and *y*_*i*_ and *y′*_*i*_∈{0, 1} are the sample labels. Train and validate the constructed network model on the two sets for *N* epochs.

Step 2. Search for the samples in *V* that are erroneously classified more than five times in the last 10 epochs, termed $V^{\prime }=\left\{\left(x_{m_{1}}^{\prime },y_{m_{1}}^{\prime }\right),\left(x_{m_{2}}^{\prime },y_{m_{2}}^{\prime }\right),\ldots ,\left(x_{m_{k}}^{\prime },y_{m_{k}}^{\prime }\right)\right\}$, and search for the samples in *T* that are correctly classified in each of the last 10 epochs, termed $T^{\prime }=\left\{\left(x_{n_{1}},y_{n_{1}}\right),\left(x_{n_{2}},y_{n_{2}}\right),\ldots ,\left(x_{n_{l}},y_{n_{l}}\right)\right\}$.

Step 3. Randomly select [*k*/2] samples from *V′*, termed *V*_*change*_, and [*k*/2] samples from *T′*, termed *T*_*change*_ (if [*k*/2] is larger than *l*, then randomly select *l* samples from *V′* and *T′*). Then, construct a training set *T*_*new*_ and a validation set *V*_*new*_ by exchanging the samples of *T* and *V* that are contained in *T*_*change*_ and *V*_*change*_.

Step 4. Retrain the network model on the two sets *T*_*new*_ and *V*_*new*_, repeat step 3 and step 4 *M* (*M* was set to 2 in this study) times, and obtain the final training and validation sets *T*_*final*_ and *V*_*final*_. Then, reinitialize the neural network and perform training and validation on *T*_*final*_ and *V*_*final*_.

#### Evaluation metrics and methods

In this study, the widely used accuracy (Acc), sensitivity (Sens), specificity (Spec), precision (Prec), F1 score, and MCC criteria were applied to evaluate the performance of the models (see Note 4 for the definitions of these criteria). To evaluate the effectiveness of the models, 5-fold cross-validation and independent testing were employed on multiple datasets. For the 5-fold cross-validation, we randomly divided all the samples into five sets of equal size, among which four were used for training and validation (the training-validation ratio was 4:1), and the remaining set was used for testing. This process was repeated five times in such a way that each of the five sets was used once for testing, and the final performance was obtained by averaging the performance achieved across all five sets.

## Data Availability

The data that support the findings of this study are available from the lead contact upon reasonable request. The authors declare that all other data supporting the findings of this study are available within the paper and its supplemental information files. TriNet is deployed on our web server: http://liulab.top/TriNet/server. All original code has been deposited at Zenodo under https://doi.org/10.5281/zenodo.7556870 and is publicly available as of the date of publication.

## Supplemental information

Document S1. Notes S1–S4, Figures S1–S13, and Tables S1–S9

Document S2. Article plus supplemental information
