# Supplementary material for: TriNet: A tri-fusion neural network for the prediction of anticancer and antimicrobial peptides
Source: Patterns (N Y). 2023 Feb 28;4(3):100702. doi: 10.1016/j.patter.2023.100702 (PMC10028424; doi:10.1016/j.patter.2023.100702)
Supplement: Document S1. Notes S1–S4, Figures S1–S13, and Tables S1–S9 [file mmc1.pdf]

Patterns, Volume *4*

Supplemental information

TriNet: A tri-fusion neural network for the prediction of anticancer and antimicrobial peptides

Wanyun Zhou, Yufei Liu, Yingxin Li, Siqi Kong, Weilin Wang, Boyun Ding, Jiyun Han, Chaozhou Mou, Xin Gao, and Juntao Liu

Supplemental Information

<u>Supplemental Notes</u>

Supplementary Note 1: Training ACP-DL by using TVI

We tested the performance differences of the ACP-DL model by randomly separating the training and validation sets 10 times as we did in the main text, and the results showed that the performance differences between the two separations were 6.1%, 8.0%, 8.2%, 6.0%, 6.0%, and 0.13 in terms of the accuracy, sensitivity, specificity, precision, F1-score, and MCC metrics, respectively, on the ACP740 dataset (see Fig. S1). On the ACPmain dataset, the differences were 4.7%, 4.7%, 6.4%, 5.2%, 4.1%, and 0.093, respectively (see Fig. S2). Then, we trained the ACP-DL model by using the new TVI training method, and the results showed that the TVI training method performed better than traditional random training, with average improvements of 2.1%, 1.6%, 2.7%, 2.2%, 1.9%, and 0.053 in terms of the accuracy, sensitivity, specificity, precision, F1-score, and MCC metrics, respectively, on the ACP740 dataset (see Fig. S1). On the ACPmain dataset, the average improvements were 2.6%, 5.9%, 4.1%, 2.3%, 3.5%, and 0.054, respectively (see Fig. S2). Moreover, the largest improvement rates in terms of the accuracy, sensitivity, specificity, precision, F1-score, and MCC metrics were 7.6%, 6.2%, 13.5%, 9.2%, 6.2%, and 18.9%, respectively, on the ACP740 dataset and 5.0%, 9.2%, 8.0%, 7.0%, 6.1%, 18.2%, respectively, on the ACPmain dataset.

Supplementary Note 2: Training MHCNN by using TVI

We also tested the performance differences of the MHCNN model by randomly separating the training and validation sets 10 times, and the results showed that the performance differences between the two separations were 6.8%, 11.8%, 16.7%, 12.1%, 7.0%, and 0.13 in terms of the accuracy, sensitivity, specificity, precision, F1-score, and MCC metrics, respectively, on the ACP740 dataset (see Fig. S3). On the ACPmain dataset, the differences were 2.6%, 15.2%, 12.3%, 6.7%, 6.4% and 0.056, respectively (see Fig. S4). Then, we trained the ACP-DL model by using the new training method TVI, and the results showed that the training method TVI performed better than traditional random training, with average improvement rates of 1.5%, 2.1%, 0.8%, 0.9%, 1.5%, and 3.6% in terms of the accuracy, sensitivity, specificity,

precision, F1-score, and MCC metrics, respectively, on the ACP740 dataset (see Fig. S3). On the ACPmain dataset, the average improvement rates were 2.4%, 2.5%, 4.6%, 3.7%, 2.0% and 9.1%, respectively (see Fig. S4). Moreover, the largest improvement rates in terms of the accuracy, sensitivity, specificity, precision, F1-score, and MCC metrics were 4.9%, 10.0%, 8.2%, 7.5%, 5.6%, and 11.6%, respectively, on the ACP740 dataset and 4.7%, 5.8%, 10.3%, 7.2%, 4.7% and 17.5%, respectively, on the ACPmain dataset.

Supplementary Note 3: Original DCGR method

The original DCGR method was developed for similarity analysis of protein sequences, and the process can be summarized in the following three steps.

Step 1. Rank the 20 amino acids according to their physicochemical properties and then map them on a unit circle uniformly with the following equation:

$$\emptyset(X_i) = (cos\frac{2\pi i}{20}, sin\frac{2\pi i}{20}) \quad i = 1,2...20$$

where X_i represents 20 amino acids that form the protein sequences.

Step 2. Construct the CGR curve for each protein sequence. The coordinate of the *i*-th amino acid s_i in the unit circle is

$$\varphi(s_i) = \frac{1}{2}\big(\varphi(s_{i-1}) + \emptyset(s_i)\big), i = 1,2...N, \varphi(s_0) = (0,0)$$

where *N* represents the length of the protein sequence. As 158 physicochemical properties were selected from the AAindex, the 20 amino acids were arranged 158 times on the unit circle for each protein sequence, and finally, 158 different CGR curves were obtained.

Step 3. The unit circle corresponding to each of the CGR curves can be divided into four segments based on the four quadrants. Then, the Euclidean distances between all points within each segment were calculated to form 4 distance matrices. For each of the 158 CGR curves, a 4-dimensional vector can be obtained by selecting the leading eigenvalues of each distance matrix.

Supplementary Note 4: Definitions of criteria used in this paper

We used the widely used accuracy (Acc), sensitivity (Sens), specificity (Spec), F1-score, precision (Prec) and Matthews correlation coefficient (Mcc) metrics to evaluate our model. Each metric defined as follows:

$$Acc = \frac{TP + TN}{TP + FN + TN + FP}$$

$$Sens = \frac{TP}{TP + FN}$$

$$Spec = \frac{TN}{TN + FP}$$

$$Prec = \frac{TP}{TP + FP}$$

$$F1 - score = \frac{2 \times Prec \times Sens}{Prec + Sens}$$

$$Mcc = \frac{TP \times TN - FP \times FN}{\sqrt{(TP + FP) \times (TP + FN) \times (TN + FP) \times (TN + FN)}}$$

where TP is true positive, FP is false positive, TN is true negative, and FN is false negative.

Supplemental Figures

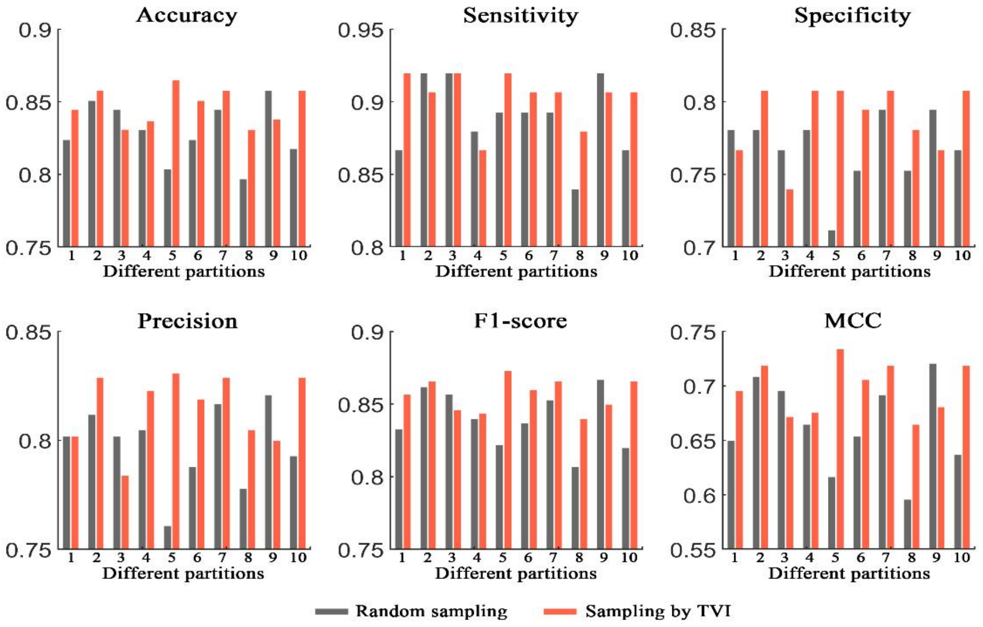

Figure S1. Performance comparison between the traditional training approach and the new TVI method on the ACP740 dataset for the ACP-DL model.

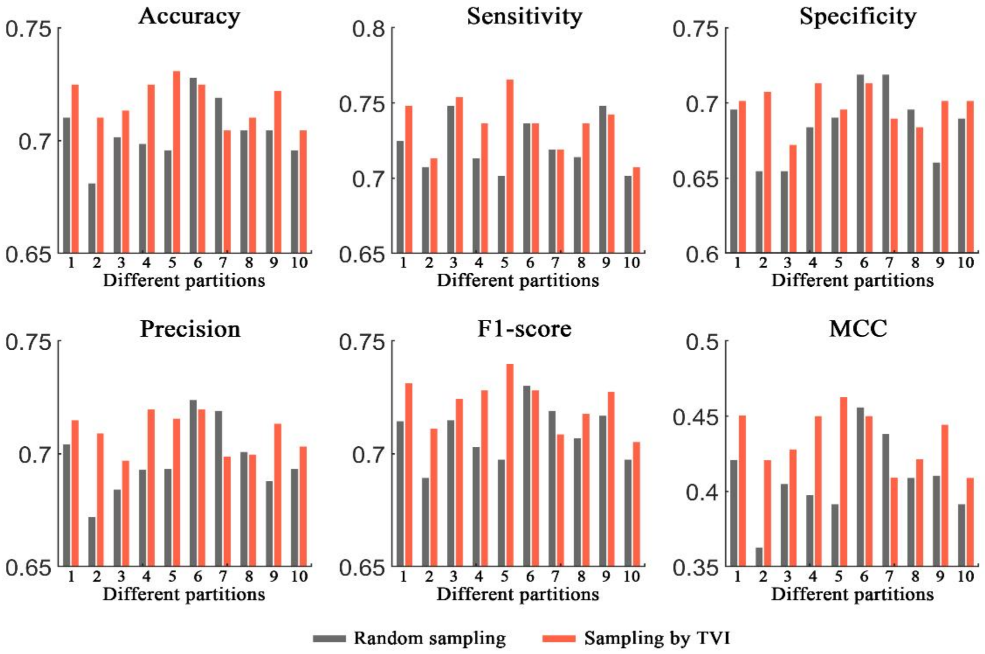

Figure S2. Performance comparison between the traditional training approach and the new TVI method on the ACPmain dataset for the ACP-DL model.

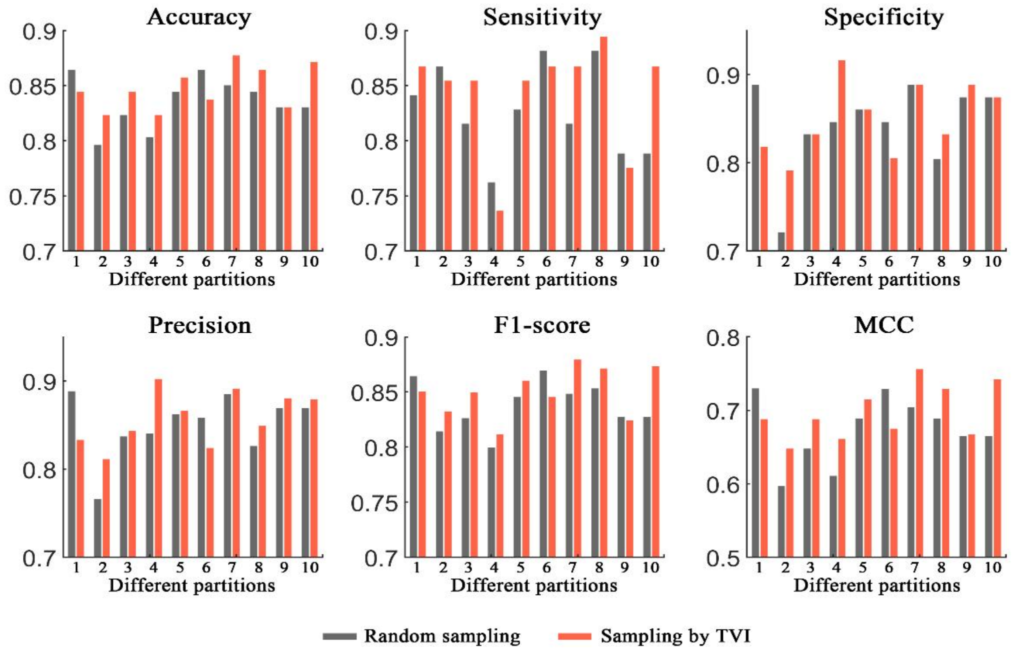

Figure S3. Performance comparison between the traditional training approach and the new TVI method on the ACP740 dataset for the MHCNN model.

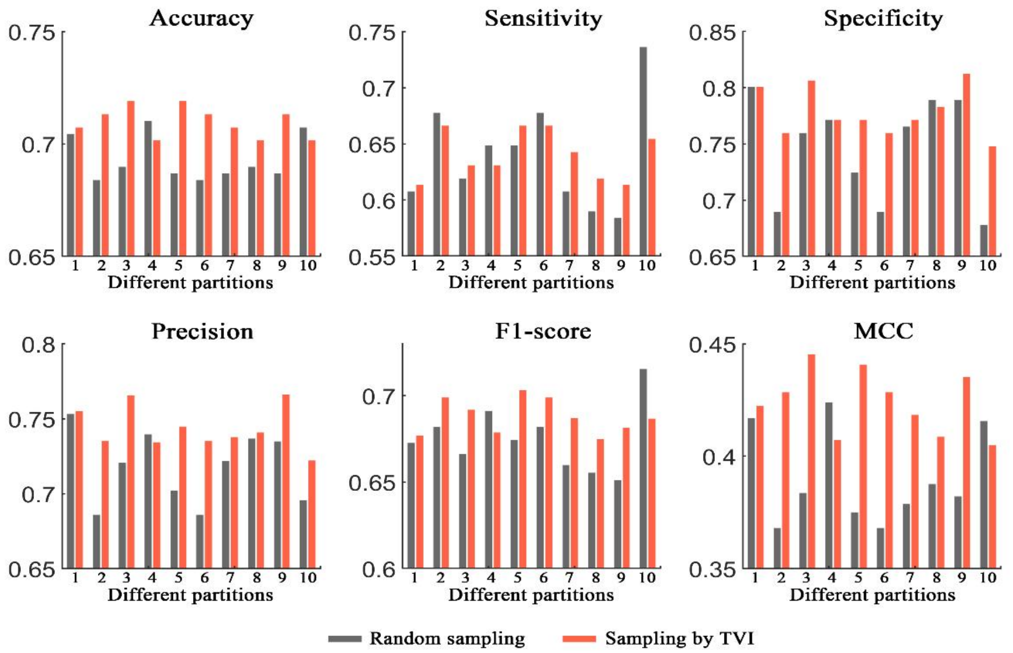

Figure S4. Performance comparison between the traditional training approach and the new TVI method on the ACPmain dataset for the MHCNN model.

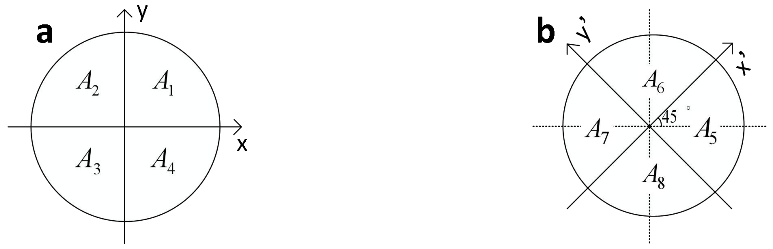

Figure S5. Calculation of the distance matrices of the improved DCGR method. a) The four distance matrices are calculated based on the four quadrants of the coordinate system. b) The four new distance matrices are calculated based on four new quadrants by rotating the coordinate axis by 45 degrees.

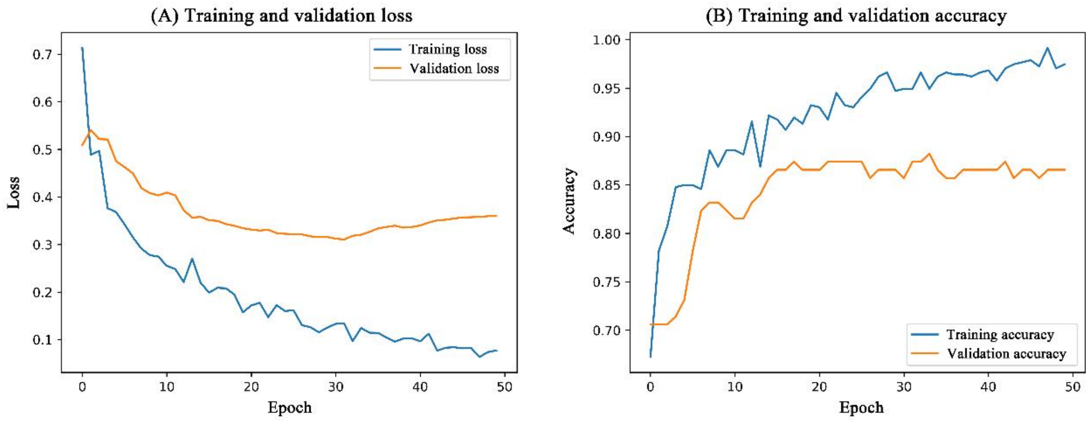

Figure S6. Learning curves on the ACP740 dataset. (A) The training loss and validation loss. (B) The training accuracy and validation accuracy.

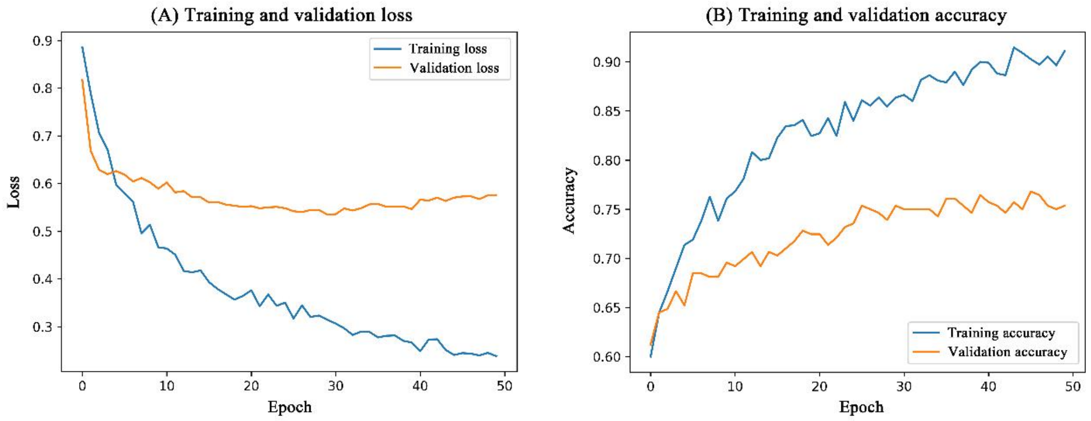

Figure S7. Learning curves on the ACPmain dataset. (A) The training loss and validation loss. (B) The training accuracy and validation accuracy.

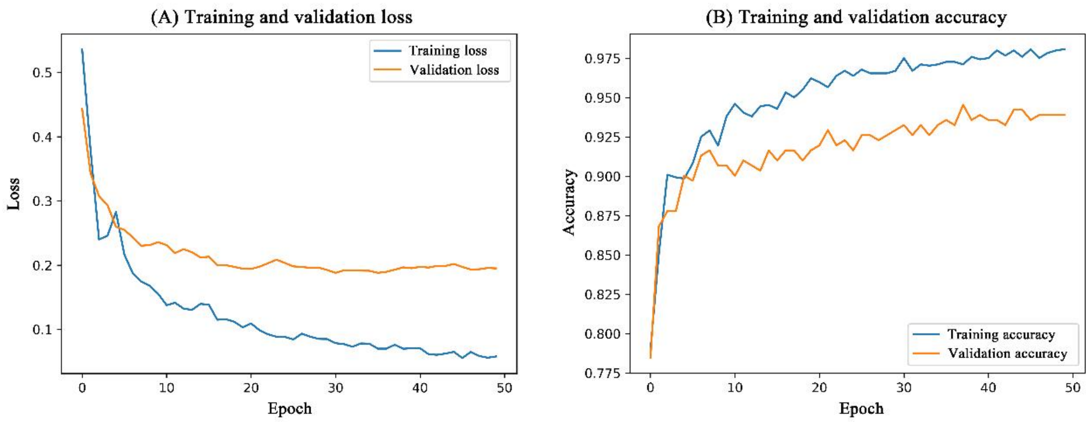

Figure S8. Learning curves on the ACPalt dataset. (A) The training loss and validation loss. (B) The training accuracy and validation accuracy.

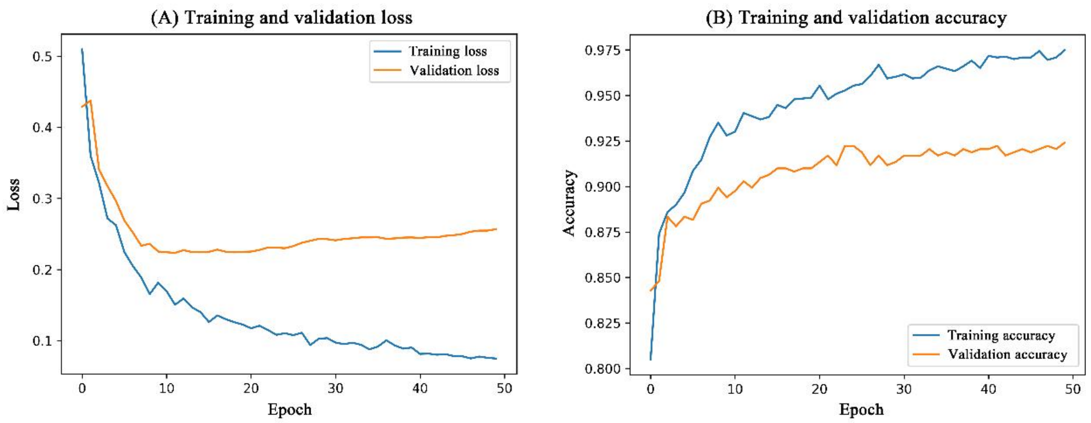

Figure S9. Learning curve on Xiao's dataset. (A) The training loss and validation loss. (B) The training accuracy and validation accuracy.

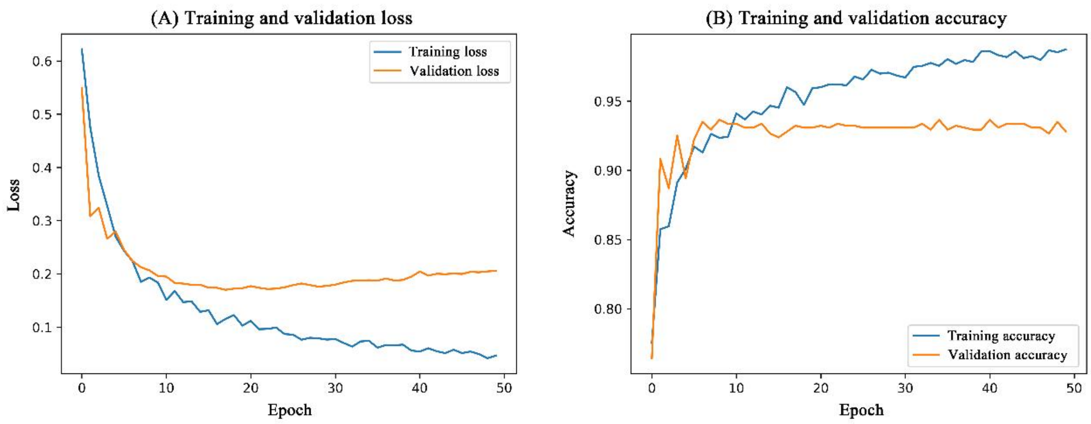

Figure S10. Learning curve on the DAMP dataset. (A) The training loss and validation loss. (B) The training accuracy and validation accuracy.

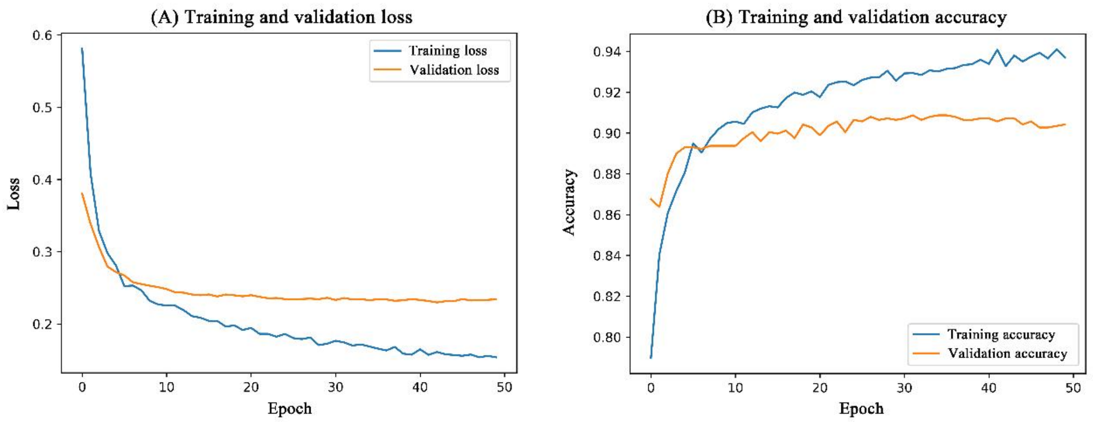

Figure S11. Learning curve on the AMPlify dataset. (A) The training loss and validation loss. (B) The training accuracy and validation accuracy.

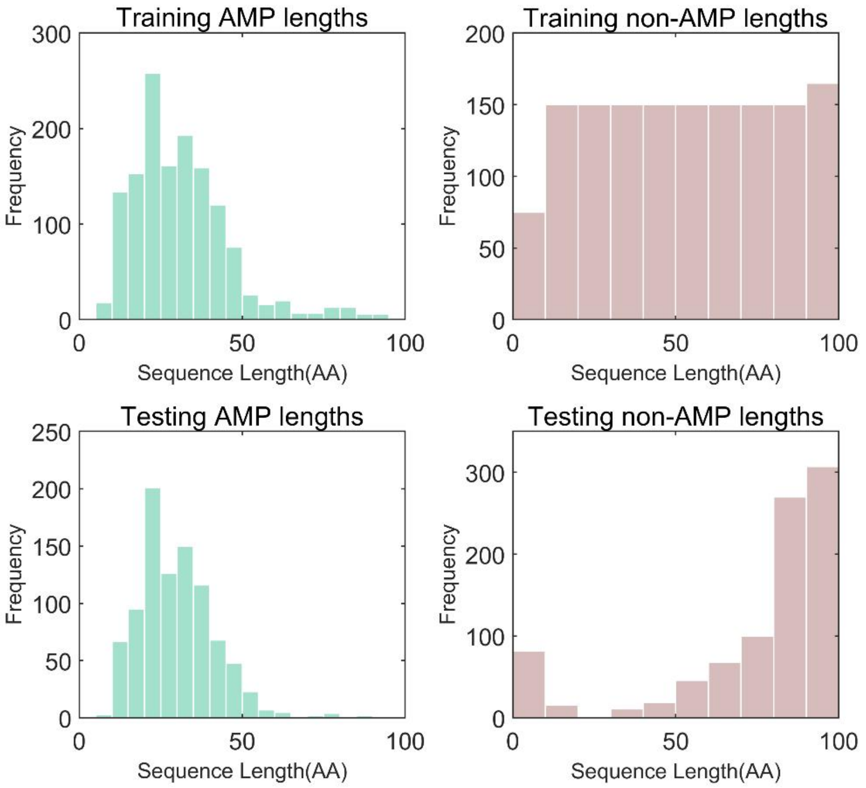

Figure S12. Sequence length distributions of AMPs (upper left) and non-AMPs (upper right) in the original Xiao training dataset and AMPs (left bottom) and non-AMPs (right bottom) in the original Xiao independent testing set.

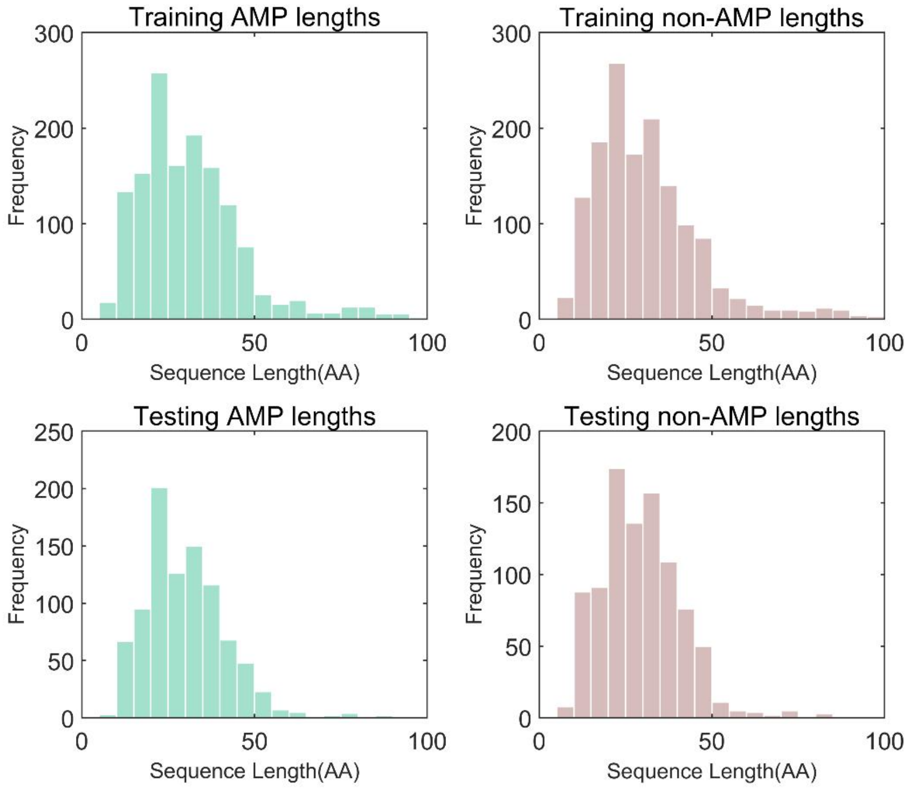

Figure S13. Sequence length distributions of AMPs (upper left) and non-AMPs (upper right) in Xiao's training dataset after our adjustments and AMPs (left bottom) and non-AMPs (right bottom) in Xiao's independent test dataset after our adjustments.

Supplemental Tables

Table S1. Comparison of the performance differences between the traditional training method and the new TVI training method for the TriNet, ACP-DL, and MHCNN models.

Model	Datasets	Training method	Acc(%)	Sens(%)	Spec(%)	Prec(%)	F1-score(%)	Mcc
TriNet	ACP740	random sampling	4.7	5.3	11.0	9.5	3.9	0.098
		sampling by TVI	**2.7**	**5.3**	**6.8**	**5.8**	**2.6**	**0.055**
	ACPmain	random sampling	4.7	7.6	8.8	5.9	4.7	0.094
		sampling by TVI	**2.9**	**6.4**	**5.3**	**3.4**	**3.7**	**0.059**
	Xiao Dataset	random sampling	1.6	0.3	3.2	2.8	1.5	0.031
		sampling by TVI	**0.8**	**0.3**	**1.5**	**1.4**	**0.8**	**0.015**
ACP-DL	ACP740	random sampling	6.1	8.0	8.2	6.0	6.0	12.5
		sampling by TVI	**3.4**	**5.3**	**6.8**	**4.7**	**3.3**	**6.9**
	ACPmain	random sampling	4.7	**4.7**	6.4	5.2	4.1	0.093
		sampling by TVI	**2.6**	5.9	**4.1**	**2.3**	**3.5**	**0.054**
MHCNN	ACP740	random sampling	6.8	**11.8**	16.7	12.1	7.0	13.3
		sampling by TVI	**5.4**	15.8	**12.5**	**9.1**	**6.8**	**10.8**

ACPmain	random sampling	2.6	15.2	12.3	6.7	6.4	0.056
	sampling by TVI	**1.8**	**5.3**	**6.4**	**4.4**	**2.9**	**0.040**

Table S2. Performance comparison of the original DCGR method (4 parts) and the improved DCGR method (8 parts) on three datasets.

Dataset	Method	Acc(%)	Sens(%)	Spec(%)	Prec(%)	F1-score(%)	Mcc
ACP740	4-parts DCGR	85.3	86.4	84.1	85.1	85.7	0.71
	8-parts DCGR	**87.7**	**88.3**	**87.8**	**88.2**	**88.2**	**0.76**
ACPmain	4-parts DCGR	70.2	71.9	68.4	69.5	70.7	0.40
	8-parts DCGR	**76.6**	**79.5**	**73.7**	**75.1**	**77.3**	**0.53**
Xiao Dataset	4-parts DCGR	94.6	98.9	90.3	91.1	94.8	0.90
	8-parts DCGR	**96.6**	**94.7**	**94.9**	**96.6**	**93.2**	**0.93**

Table S3. Performance comparison of different feature combinations on three datasets.

Dataset	Component	Acc(%)	Sens(%)	Spec(%)	Prec(%)	F1-score(%)	Mcc
ACP740	TriNet	**87.7**	**88.3**	**87.8**	**88.2**	**88.2**	**0.76**
	-no DCGR	82.2	77.7	86.7	85.9	81.6	0.65
	-no PSSM	85.0	85.2	84.8	85.5	85.3	0.71
	-no PCPE	86.2	87.2	85.2	86.1	86.6	0.73
ACPmain	TriNet	**76.6**	79.5	**73.7**	**75.1**	**77.3**	**0.53**
	-no DCGR	64.3	78.9	49.7	61.1	68.9	0.30
	-no PSSM	71.6	**80.1**	63.2	68.5	73.8	0.44
	-no PCPE	73.1	74.9	71.3	72.3	73.6	0.46
Xiao Dataset	TriNet	**96.6**	**98.5**	**94.7**	**94.9**	**96.6**	**0.93**
	-no DCGR	86.1	91.0	81.3	82.9	86.7	0.73
	-no PSSM	95.1	98.2	92.0	92.4	95.2	0.90
	-no PCPE	95.8	98.5	93.2	93.5	95.9	0.92

Table S4. Performance comparison of the extracted features in this study based on XGBoost and existing network models on the ACP740 dataset.

Model	Acc(%)	Sens(%)	Spec(%)	Prec(%)	F1-score(%)	Mcc
ACP-DL	81.5	82.6	80.6	82.4	82.5	0.63
MHCNN	83.1	85.4	81.1	82.8	84.1	0.67
iAcp-DRLF	80.7	86.7	72.2	78.7	82.5	0.61
CL-ACP	83.8	82.9	84.8	85.2	84.0	0.68
DeepACPred	85.0	85.3	85.0	85.6	85.5	0.71
DCGR+PSSM+ PCPE+XGBoost	**85.4**	**85.4**	**85.9**	**85.7**	**85.5**	**0.71**

Table S5. Performance comparison of the extracted features in this study based on XGBoost and existing network models on the ACPmain independent dataset.

Model	Acc(%)	Sens(%)	Spec(%)	Prec(%)	F1-score(%)	Mcc
ACP-DL	71.4	73.1	69.6	70.6	**74.3**	0.43
MHCNN	68.4	67.8	69.0	68.6	68.2	0.37
AntiCP 2.0	72.3	69.4	75.2	**73.8**	71.5	0.45
iACP-DRLF	**74.3**	**75.4**	73.1	73.7	74.6	**0.49**
DCGR+PSSM +PCPE+XGB oost	74.0	73.3	**75.4**	72.5	72.9	0.48

Table S6. Performance comparison of the extracted features in this study based on XGBoost and existing network models on Xiao's independent test set

Model	Acc(%)	Sens(%)	Spec(%)	Prec(%)	F1-score(%)	Mcc
DNN	**93.9**	96.6	**91.2**	**91.7**	**94.1**	**0.88**
APIN	93.9	97.3	90.4	91.1	94.1	0.88
ACEP	88,9	95.7	82.0	84.1	89.5	0.78

CAMP-RF	89.4	97.7	81.0	83.7	90.2	0.80
CAMP-SVM	90.2	96.5	83.9	85.7	90.8	0.81
CAMP-ANN	88.1	93.2	83.0	84.6	88.7	0.77
DCGR+PSSM+ PCPE+XGBoost	92.3	**98.9**	85.7	87.3	92.8	0.85

Table S7. Performance comparison of different kernel sizes on three datasets.

Dataset	kernel size	Acc(%)	Sens(%)	Spec(%)	Prec(%)	F1-score(%)	Mcc
ACP740	2× 2	84.9	84.8	84.9	85.4	85.1	0.70
	3 × 3	85.7	85.4	86.1	86.3	85.8	0.71
	5 × 5	83.9	84.3	83.5	84.1	84.2	0.68
	1 × 8	**87.7**	**88.3**	**87.8**	**88.2**	**88.2**	**0.76**
ACPmain	2× 2	71.1	71.9	70.2	70.7	71.3	0.42
	3 × 3	71.9	71.9	71.9	71.9	71.9	0.44
	5 × 5	73.7	76.0	71.3	72.6	74.3	0.47
	1 × 8	**76.6**	**79.5**	**73.7**	**75.1**	**77.3**	**0.53**
Xiao Dateset	2× 2	95.7	**99.0**	92.3	92.8	**95.8**	0.92
	3 × 3	95.3	97.5	93.0	93.3	95.4	0.91
	5 × 5	92.5	93.7	91.2	91.4	92.5	0.85
	1 × 8	**96.6**	94.7	**94.9**	**96.6**	93.2	**0.93**

Table S8. Performance comparison of different channel attention mechanisms on three datasets.

Dataset	channel attention	Acc(%)	Sens(%)	Spec(%)	Prec(%)	F1-score(%)	Mcc
ACP740	SENet	85.5	85.4	85.7	86.2	85.8	0.71
	CBAM	85.1	87.0	83.3	84.5	85.7	0.70
	CAM	**87.7**	**88.3**	**87.8**	**88.2**	**88.2**	**0.76**
ACPmain	SENet	72.2	72.5	71.9	72.1	72.3	0.44
	CBAM	72.2	71.3	73.1	72.6	71.9	0.44

	CAM	**76.6**	**79.5**	**73.7**	**75.1**	**77.3**	**0.53**
	SENet	94.6	97.9	91.2	91.8	94.7	0.89
Xiao Dataset	CBAM	96.1	**99.0**	93.2	93.5	96.2	0.92
	CAM	**96.6**	98.5	**94.7**	**94.9**	**96.6**	**0.93**

Table S9. Performance comparison of different numbers of heads in the self-attention mechanism on three datasets.

Dataset	head	Acc(%)	Sens(%)	Spec(%)	Prec(%)	F1-score(%)	Mcc(%)
	1	**87.7**	**88.3**	**87.8**	**88.2**	**88.2**	**0.76**
ACP740	2	86.2	86.7	85.7	86.3	86.5	0.73
	4	85.5	86.4	84.6	85.4	85.9	0.71
	1	**76.6**	**79.5**	**73.7**	**75.1**	**77.3**	**0.53**
ACPmain	2	73.1	74.3	71.9	72.6	73.4	0.46
	4	71.9	70.2	73.7	72.7	71.4	0.44
	1	**96.6**	**98.5**	**94.7**	**94.9**	**96.6**	**0.93**
Xiao Dataset	2	96.5	98.4	94.7	94.9	96.6	0.93
	4	96.5	98.4	94.7	94.9	96.6	0.93
